# Intensification of Diabetes Medications at Hospital Discharge and Clinical Outcomes in Older Adults in the Veterans Administration Health System

**DOI:** 10.1001/jamanetworkopen.2021.28998

**Published:** 2021-10-21

**Authors:** Timothy S. Anderson, Alexandra K. Lee, Bocheng Jing, Sei Lee, Shoshana J. Herzig, W. John Boscardin, Kathy Fung, Anael Rizzo, Michael A. Steinman

**Affiliations:** 1 Division of General Medicine, Beth Israel Deaconess Medical Center, Boston, Massachusetts; 2 Harvard Medical School, Boston, Massachusetts; 3 San Francisco Veterans Affairs Medical Center, San Francisco, California; 4 Division of Geriatrics, University of California, San Francisco

## Abstract

**Importance:**

Transient elevations of blood glucose levels are common in hospitalized older adults with diabetes and may lead clinicians to discharge patients with more intensive diabetes medications than they were using before hospitalization.

**Objective:**

To investigate outcomes associated with intensification of outpatient diabetes medications at discharge.

**Design, Setting, and Participants:**

This retrospective cohort study assessed patients 65 years and older with diabetes not taking insulin who were hospitalized in the Veterans Health Administration Health System between January 1, 2011, and September 28, 2016, for common medical conditions. Data analysis was performed from January 1, 2020, to March 31, 2021.

**Exposure:**

Discharge with intensified diabetes medications, defined as filling a prescription at hospital discharge for a new or higher-dose medication than was being used before hospitalization. Propensity scores were used to construct a matched cohort of patients who did and did not receive diabetes medication intensifications.

**Main Outcomes and Measures:**

Coprimary outcomes of severe hypoglycemia and severe hyperglycemia were assessed at 30 and 365 days using competing risk regressions. Secondary outcomes included all-cause readmissions, mortality, change in hemoglobin A_1c_ (HbA_1c_) level, and persistent use of intensified medications at 1 year after discharge.

**Results:**

The propensity-matched cohort included 5296 older adults with diabetes (mean [SD] age, 73.7 [7.7] years; 5212 [98.4%] male; and 867 [16.4%] Black, 47 [0.9%] Hispanic, 4138 [78.1%] White), equally split between those who did and did not receive diabetes medication intensifications at hospital discharge. Within 30 days, patients who received medication intensifications had a higher risk of severe hypoglycemia (hazard ratio [HR], 2.17; 95% CI, 1.10-4.28), no difference in risk of severe hyperglycemia (HR, 1.00; 95% CI, 0.33-3.08), and a lower risk of death (HR, 0.55; 95% CI, 0.33-0.92). At 1 year, no differences were found in the risk of severe hypoglycemia events, severe hyperglycemia events, or death and no difference in change in HbA_1c_ level was found among those who did vs did not receive intensifications (mean postdischarge HbA_1c_, 7.72% vs 7.70%; difference-in-differences, 0.02%; 95% CI, −0.12% to 0.16%). At 1 year, 48.0% (591 of 1231) of new oral diabetes medications and 38.5% (548 of 1423) of new insulin prescriptions filled at discharge were no longer being filled.

**Conclusions and Relevance:**

In this national cohort study, among older adults hospitalized for common medical conditions, discharge with intensified diabetes medications was associated with an increased short-term risk of severe hypoglycemia events but was not associated with reduced severe hyperglycemia events or improve HbA_1c_ control. These findings indicate that short-term hospitalization may not be an effective time to intervene in long-term diabetes management.

## Key Points

**Question:**

What is the association between intensification of outpatient diabetes medications at hospital discharge and clinical outcomes in older adults hospitalized for common medical conditions?

**Findings:**

In this cohort study of 5296 propensity-matched older veterans with diabetes who were hospitalized for common medical conditions, discharge with intensified diabetes medication was associated with an increased risk of severe hypoglycemia within 30 days and was not associated with a reduction in severe hyperglycemia events or hemoglobin A_1c_ level at 1 year.

**Meaning:**

These findings indicate that short-term hospitalization may not be an effective time to intervene in long-term diabetes management.

## Teaser

This cohort study of older US veterans evaluated the association between intensification of home diabetes medications at hospital discharge and postdischarge outcomes.

## Introduction

Modification of older adults’ home medications during short-term hospitalization is common. Changes to home medications may be temporary in response to acute illness or may reflect planned changes to management of chronic disease.^1,2,3,4,5^ A particularly common scenario is adjustment of diabetes medications.^5,6^ During hospitalization for acute illness, older adults with diabetes may experience fluctuating blood glucose control, driven by changes in eating patterns, medication exposures, and catecholamine surges. As a result, hospitalization is a high-risk time for serious hypoglycemia and hyperglycemia events.^7,8,9,10,11^ Practice guidelines advise broader ranges for inpatient blood glucose levels than are recommended in the outpatient setting and recommend stopping the use of home oral agents and initiating the use of short-term insulin in many clinical scenarios.^12,13,14^

Safe diabetes management for older adults requires careful balancing of the short-term risks of medication-induced hypoglycemia^15,16,17^ with the long-term benefits of blood glucose control.^18,19^ Transient elevations in blood glucose during hospitalization likely have little long-term significance, yet these increases commonly precipitate intensification of home regimens, including discharging patients with new insulin or oral agents.^5^ For patients with uncontrolled diabetes, hospitalization could be an opportune time to address hyperglycemia and set patients on the path toward improved chronic disease control. However, the posthospitalization period is also a particularly high-risk time for adverse drug events and medication errors. Although clinical trials and guidelines have helped inform long-term diabetes treatment strategies for ambulatory older adults, this evidence does not reflect the perihospitalization period, during which time older adults have an increased susceptibility to adverse drug events^20,21^ and may not be generalizable to hospitalized older adult populations, who face greater frailty and more limited life expectancy than clinical trial participants.^22,23^

Because the clinical outcomes associated with diabetes medication intensifications made at hospital discharge are unknown, we conducted a retrospective cohort study of older adults with diabetes who were hospitalized in the national Veterans Health Administration (VHA) health system for common noncardiac conditions. We evaluated the association between intensification of home diabetes medications at hospital discharge and postdischarge outcomes, including severe hypoglycemia and hyperglycemia events, mortality, hemoglobin A_1c_ (HbA_1c_) control at 1 year, and persistent use of discharge medications at 1 year after discharge.

## Methods

We conducted a retrospective cohort study using national inpatient and outpatient VHA pharmacy and clinical data merged with VHA and Medicare claims data from 2009 to 2018. This research was approved by the institutional review boards of the San Francisco Veterans Affairs Medical Center and the University of California, San Francisco. A waiver of informed consent was obtained because administrative data were used and all data were deidentified. Data analysis was performed from January 1, 2020, to March 31, 2021. This study followed the Strengthening the Reporting of Observational Studies in Epidemiology (STROBE) reporting guideline.

### Study Population

The study population consisted of adults with diabetes 65 years and older who were admitted to a VHA hospital between January 1, 2011, and September 28, 2016, for common medical conditions and discharged to the community setting (eFigure 1 in the Supplement). Diabetes was defined by the presence of 2 outpatient diagnoses or any hospital discharge diagnosis of diabetes in the 2 years that preceded the index hospitalization using previously validated algorithms.^24,25^ Because diagnosis-based algorithms may capture patients with a history of diabetes or currently being evaluated for diabetes, to enhance specificity we examined only patients who were taking a diabetes medication before hospitalization or had an HbA_1c_ level greater than 6.5% (to convert to proportion of total hemoglobin, multiply by 0.01) in the year before hospitalization.

Conditions were identified by primary discharge diagnosis code grouped by Clinical Classification Software categories and included the following: acute coronary syndrome, arrhythmia, asthma, chest pain, chronic obstructive pulmonary disease, coronary artery disease, conduction disorders, heart failure, heart valve disorders, pneumonia, sepsis, skin infection, stroke, transient ischemic attack, and urinary tract infection. These conditions were chosen because they are among the most common medical discharge diagnoses for older adults and their short-term management does not typically require intensification of outpatient diabetes medications. Patients discharged with a secondary discharge diagnosis of diabetic ketoacidosis or nonketotic hyperglycemic-hyperosmolar coma were excluded because these conditions typically necessitate an immediate change in diabetes treatment.

To ensure accurate classification of medication use, we excluded patients likely to receive medications outside the VHA, including patients who received more than 20% of their outpatient care outside the VHA, patients admitted from skilled nursing facilities, and patients who had been hospitalized in the 30 days that preceded the index hospitalization.^26^ Patients enrolled in hospice were excluded given differing goals of care. Because instructions to modify insulin dosing are infrequently accompanied by a new prescription, dosing changes cannot be accurately assessed using pharmacy databases; thus, we limited our study to patients not using insulin before hospitalization.

### Exposure

We compared patients discharged with intensified diabetes medication regimens to those discharged without intensifications. Intensifications were defined as newly prescribed diabetes medications that were not being used before hospitalization and medications present on admission for which a discharge prescription was filled for a dose increase of more than 20%. Intensifications were ascertained based on VHA pharmacy dispensing data using previously published methods, which included medications filled within 2 days before to 2 days after discharge.^26,27^ We examined all medication classes in use during the study period: biguanides, sulfonylureas, thiazolidinediones, α-glucosidase inhibitors, dipeptidyl peptidase 4 inhibitors, meglitinides, glucagon-like peptide 1 (GLP-1) agonists, sodium-glucose cotransporter 2 (SGLT2) inhibitors, and insulins.

### Outcomes

The 2 primary outcomes were chosen a priori to assess possible benefits and harms of diabetes medication intensification: severe hyperglycemia events and severe hypoglycemia events. Primary outcomes were examined at 30 days to assess immediate outcomes and at 365 days to assess longer term outcomes. On the basis of prior studies,^15,28,29^ primary outcomes were defined as a composite of emergency department (ED) visits, observation stays, and hospitalizations for severe hypoglycemia and severe hyperglycemia (eTable 1 in the Supplement). Secondary outcomes included all-cause readmissions at 30 and 365 days, mortality at 30 and 365 days, change in HbA_1c_ at 1 year, and persistent use of diabetes medication prescriptions filled at discharge at 1 year.

### Statistical Analysis

We used propensity score approaches that involved multiple steps. First, a logistic regression model was developed to estimate the propensity of receiving a medication intensification at discharge. Covariates were derived from variables examined in prior diabetes treatment trials^13,30^ and according to clinical expertise^18^ and included demographic characteristics, comorbidities,^31^ prehospitalization and hospital vital signs, laboratory values, health care use, and medications (eTable 2 in the Supplement). Missing data were imputed using the fully conditional specification method and 20 imputation sets. One-to-one nearest neighbor matching without replacement was performed and covariate balance between groups was assessed using standardized mean differences.^32,33^

Second, within propensity score–matched groups, survival analyses were conducted for hyperglycemia, hypoglycemia, mortality, and readmission outcomes using Cox proportional hazards regression models for mortality and Fine and Gray proportional subdistribution hazards models for all other outcomes to account for the competing risk of death.^34^ For all models, SEs accounted for clustering of patients within hospitals. To aid in interpretation of subdistribution hazard models, unadjusted event rates are presented for each group.

Third, within propensity score–matched groups, the change in HbA_1c_ at 1 year after discharge was estimated using a difference-in-differences approach. Linear regression models were used to estimate the change in HbA_1c_ level associated with discharge with intensified diabetes medications after subtracting the background change among patients who did not receive medication intensifications.^35,36^

Fourth, for the unmatched cohort, we examined persistence to diabetes medications filled at discharge during the subsequent year. We examined diabetes medication prescriptions filled at discharge, including new medication prescriptions and prescription fills of admission medications at higher, lower, or the same doses. For each diabetes medication filled at discharge, we calculated persistence as the number of days between the discharge fill and the last refill for the same or greater dose plus the days supplied by the latest refill.^37^ We constructed Kaplan-Meier curves and used the log-rank test to examine differences in persistence by type of fill: continuation, dose increase, dose decrease, new oral medication, or new insulin.

We conducted subgroup analyses to determine the differential impact of exposure to intensified diabetes medications by prehospitalization diabetes control. We classified patients as having controlled or elevated prehospitalization HbA_1c_ levels using a threshold HbA_1c_ of 7.5%, acknowledging the uncertainty surrounding exact HbA_1c_ targets in older adults.^18,22,38^ We then repeated propensity score matching and analyses for each baseline HbA_1c_ group separately.

Analyses were conducted using Stata software, version 14.1 (StataCorp LLC). For all analyses, we determined statistical significance using 95% CIs. A 2-sided *P* < .05 was also considered statistically significant.

## Results

The unmatched cohort included 28 198 older adults with diabetes admitted to 115 VHA hospitals (mean [SD] age, 73.7 [7.7] years; 27 710 [98.3%] male; 4160 [14.8%] Black; 394 [1.4%] Hispanic, 22 600 [80.1%] White), of whom 2768 (9.8%) received diabetes medication intensifications at discharge. Most intensifications were new insulins (n = 1423) or sulfonylureas (n = 640) (eTable 2 in the Supplement). Patients discharged with medication intensifications were younger, had higher mean prehospitalization HbA_1c_ values, higher inpatient blood glucose recordings, fewer admission medications, and longer length of stay (Table 1; eTable 3 in the Supplement).

**Table 1.** Selected Cohort Characteristics Before and After Propensity Score Matching

Characteristic^a^^,^^b^	Before propensity score matching			After propensity score matching		
	Intensified (n = 2768)	Not intensified (n = 25 430)	SMD^c^	Intensified (n = 2648)	Not intensified (n = 2648)	SMD^c^
Age, mean (SD), y	72.6 (7.3)	73.8 (7.7)	0.16	72.7 (7.3)	72.8 (7.3)	0.01
Sex, No. (%)						
Male	2721 (98.3)	24 989 (98.3)	0.00	2603 (98.3)	2609 (98.5)	0.02
Female	47 (1.7)	441 (1.7)		45 (1.7)	39 (1.5)	
Race and ethnicity, No. (%)						
Black	456 (16.5)	3704 (14.6)	0.09	426 (16.1)	441 (16.7)	0.02
Hispanic	28 (1.0)	366 (1.4)		26 (1.0)	21 (0.8)	
White	2155 (77.9)	20 445 (80.4)		2074 (78.3)	2064 (77.9)	
Other^d^	129 (4.7)	915 (3.6)		122 (4.6)	122 (4.6)	
Preadmission clinical characteristics, mean (SD)						
BMI	31.2 (6.6)	30.8 (6.5)	0.06	31.2 (6.5)	31.0 (6.7)	0.03
SBP, mm Hg	134.8 (17.9)	133.0 (17.5)	0.10	134.7 (17.9)	134.7 (17.9)	0.00
Hemoglobin A_1c_, mean (SD), %	8.0 (1.7)	7.1 (1.1)	0.68	7.9 (1.5)	7.9 (1.7)	0.02
Estimated glomerular filtration rate, mL/min/1.73 m^2^	65.5 (23.8)	67.0 (24.3)	0.06	65.5 (23.7)	65.5 (24.0)	0.00
Any hypoglycemia hospitalizations in prior year, No. (%)	38 (1.4)	211 (0.8)	0.05	36 (1.4)	53 (2.0)	0.05
Admission diabetes medication count, No. (%)						
0	1062 (38.4)	6311 (24.8)	0.31	974 (36.8)	988 (37.3)	0.04
1	1073 (38.8)	13 113 (51.6)		1046 (39.5)	1003 (37.9)	
2	547 (19.8)	5349 (21.0)		542 (20.5)	559 (21.1)	
≥3	86 (3.1)	657 (2.6)		86 (3.3)	98 (3.7)	
Admission diabetes medication classes, No. (%)^e^						
Metformin	1071 (38.7)	12 462 (49.0)	0.21	1056 (39.9)	1038 (39.2)	0.01
Sulfonylureas	1127 (40.7)	11 758 (46.2)	0.11	1108 (41.8)	1140 (43.1)	0.02
Thiazolidinediones	107 (3.9)	696 (2.7)	0.06	105 (4.0)	108 (4.1)	0.01
α-Glucosidase inhibitors	66 (2.4)	373 (1.5)	0.07	66 (2.5)	71 (2.7)	0.01
Dipeptidyl peptidase 4 inhibitors	45 (1.6)	357 (1.4)	0.02	44 (1.7)	53 (2.0)	0.03
Other classes	4 (0.1)	50 (0.2)	0.01	4 (0.2)	2 (0.1)	0.02
Preadmission health care use						
Hospitalizations in the year preceding index hospitalization, No. (%)						
0	1957 (70.7)	18 077 (71.1)	0.02	1885 (71.2)	1887 (71.3)	0.01
1	480 (17.3)	4488 (17.6)		450 (17.0)	457 (17.3)	
2	186 (6.7)	1648 (6.5)		175 (6.6)	169 (6.4)	
≥3	145 (5.2)	1217 (4.8)		138 (5.2)	135 (5.1)	
Admission medication count, mean (SD)	7.9 (4.7)	8.9 (4.9)	0.22	8.0 (4.7)	8.0 (5.0)	0.00
Index hospitalization characteristics						
Length of stay, mean (SD), d	6.6 (8.0)	5.2 (5.8)	0.20	6.5 (8.1)	6.4 (8.7)	0.01
Discharge diagnoses, No. (%)						
Arrhythmia	18 (0.7)	370 (1.5)	0.18	18 (0.7)	17 (0.6)	0.08
Asthma	22 (0.8)	124 (0.5)		21 (0.8)	19 (0.7)	
COPD	267 (9.6)	2156 (8.5)		255 (9.6)	264 (10.0)	
Chest pain	82 (3.0)	940 (3.7)		80 (3.0)	72 (2.7)	
Conduction disorders	212 (7.7)	2737 (10.8)		206 (7.8)	178 (6.7)	
Coronary artery disease	356 (12.9)	2794 (11.0)		341 (12.9)	356 (13.4)	
Acute coronary syndrome	172 (6.2)	1520 (6.0)		162 (6.1)	175 (6.6)	
Heart failure	484 (17.5)	3854 (15.2)		464 (17.5)	482 (18.2)	
Heart valve disorders	65 (2.3)	588 (2.3)		65 (2.5)	62 (2.3)	
Pneumonia	275 (9.9)	2830 (11.1)		268 (10.1)	255 (9.6)	
Sepsis	77 (2.8)	797 (3.1)		68 (2.6)	59 (2.2)	
Skin infection	246 (8.9)	2261 (8.9)		231 (8.7)	223 (8.4)	
Stroke	175 (6.3)	1320 (5.2)		164 (6.2)	197 (7.4)	
TIA	37 (1.3)	415 (1.6)		35 (1.3)	33 (1.2)	
Urinary tract infection	212 (7.7)	2139 (8.4)		205 (7.7)	199 (7.5)	
Venous thromboembolism	68 (2.5)	585 (2.3)		65 (2.5)	57 (2.2)	
Hospital blood glucose, mean (SD)						
Highest glucose, mg/dL	310.6 (110.6)	235.7 (85.3)	0.76	304.0 (105.5)	306.4 (109.4)	0.02
Lowest glucose, mg/dL	107.7 (45.2)	102.4 (32.3)	0.13	108.0 (44.2)	107.7 (43.4)	0.01
No. of hospital glucose recordings, mean (SD)	57.4 (29.5)	39.6 (22.8)	0.68	55.8 (28.3)	56.4 (30.0)	0.02
Laboratory values at discharge, mean (SD)^f^						
Glucose, mg/dL	181.8 (71.4)	156.5 (57.7)	0.39	180.7 (70.3)	181.0 (68.9)	0.00
Sodium, mEq/L	137.6 (3.2)	138.0 (3.2)	0.12	137.7 (3.2)	137.6 (3.2)	0.01
Estimated glomerular filtration rate, mL/min/1.73 m^2^	66.9 (26.3)	69.6 (26.9)	0.10	67.0 (26.2)	66.9 (26.9)	0.00
Comorbidities, No. (%)^g^						
Heart failure	1059 (38.3)	9263 (36.4)	0.04	1008 (38.1)	1019 (38.5)	0.01
Chronic angina and coronary artery disease	1457 (52.6)	13 202 (51.9)	0.01	1393 (52.6)	1434 (54.2)	0.03
Acute stroke or TIA	483 (17.4)	4173 (16.4)	0.03	454 (17.1)	494 (18.7)	0.04
COPD or asthma	1165 (42.1)	10 892 (42.8)	0.02	1117 (42.2)	1108 (41.8)	0.01
Renal disorders (including renal failure and fluid, electrolyte, and acid-base abnormalities)	1457 (52.6)	12 681 (49.9)	0.06	1376 (52.0)	1344 (50.8)	0.02
Anemia	882 (31.9)	8352 (32.8)	0.02	846 (31.9)	839 (31.7)	0.01
Cancer	544 (19.7)	5972 (23.5)	0.09	528 (19.9)	505 (19.1)	0.02
Liver disease	230 (8.3)	2217 (8.7)	0.01	218 (8.2)	226 (8.5)	0.01
Cognitive	299 (10.8)	2901 (11.4)	0.02	282 (10.6)	311 (11.7)	0.03
Substance abuse	301 (10.9)	2526 (9.9)	0.03	280 (10.6)	281 (10.6)	0.00
Skin ulcer (including decubitus ulcer)	346 (12.5)	2582 (10.2)	0.07	321 (12.1)	322 (12.2)	0.00

Abbreviations: BMI, body mass index (calculated as weight in kilograms divided by height in meters squared); COPD, chronic obstructive pulmonary disease; SBP, systolic blood pressure; SMD, standardized mean difference; TIA, transient ischemic attack.

SI conversion factors: To convert glucose to millimoles per liter, multiply by 0.0555; hemoglobin A_1c_ to proportion of total hemoglobin, multiply by 0.01; and sodium to millimoles per liter, multiply by 1.0.

^a^ Selected covariates are presented; a full list of covariates included in the propensity score is given in eTable 2 in the Supplement.

^b^ No more than 8% of any covariate was missing. Missing data were imputed using the fully conditional specification method and 20 imputation sets. Missing data included the following: BMI (n = 2282), outpatient blood pressure (n = 1594), preadmission estimated glomerular filtration rate (n = 1560), inpatient glucose (n = 2012), discharge hemoglobin (n = 1705), discharge potassium (n = 961), discharge sodium (n = 883), discharge blood urea nitrogen (n = 1337), discharge carbon dioxide (n = 1201), discharge platelets (n = 1235), and discharge estimated glomerular filtration rate (n = 1476).

^c^ Balance between the groups was assessed before and after matching by comparing SMDs for each variable for which a difference of less than 0.10 was considered to indicate adequate balance.

^d^ Other includes unknown, Asian, North American Native, and unspecified other. This categorization is drawn from Veterans Affairs and Medicare administrative records.

^e^ All medications classified using Veterans Affairs drug class coding. Combination medications were split into component parts. Topical, inhaled, otic, and optic medications were excluded.

^f^ Laboratory data collected from day of index hospitalization discharge or during index hospitalization up to 2 days before day of discharge.

^g^ Comorbidities include both secondary discharge diagnoses from index hospitalization and preadmission diagnoses from the year that preceded the index hospitalization.

The propensity-matched cohort included 5296 older adults with diabetes (mean [SD] age, 73.7 [7.7] years; 5212 [98.4%] male; and 867 [16.4%] Black, 47 [0.9%] Hispanic, 4138 [78.1%] White). A total of 2648 patients who received diabetes medication intensifications were matched to 2648 patients with a similar propensity score who did not receive intensifications (95.7% match rate). Matched groups were well balanced on propensity score distribution and baseline characteristics (standardized mean differences for all covariates, <0.1) (Table 1; eTable 3 and eFigure 2 in the Supplement).

### Primary Outcomes

In the propensity score–matched cohort, patients discharged with diabetes medication intensifications were more likely to experience a severe hypoglycemia event within 30 days of discharge than patients discharged without diabetes medication intensifications, although outcomes were rare in both groups (26/2648 [1.0%] vs 12/2648 [0.5%]; hazard ratio [HR], 2.17; 95% CI, 1.10-4.28) (Table 2). There was no difference in risk of severe hyperglycemia at 30 days (HR, 1.00; 95% CI, 0.33-3.08). No differences were found between groups in severe hyperglycemia or severe hypoglycemia at 365 days after discharge.

**Table 2.** Primary and Secondary Clinical Outcomes Associated With Receiving a Diabetes Medication Intensification at Hospital Discharge

Outcome	Patients, No. (%)		HR (95% CI)
	Intensified regimen (n = 2648)	Not intensified regimen (n = 2648)	
**Primary outcomes**			
Severe hypoglycemia			
30 d	26 (1.0)	12 (0.5)	2.17 (1.10-4.28)
365 d	83 (3.1)	76 (2.9)	1.10 (0.83-1.45)
Severe hyperglycemia			
30 d	7 (0.3)	7 (0.3)	1.00 (0.33-3.08)
365 d	34 (1.3)	35 (1.3)	0.97 (0.60-1.58)
**Secondary outcomes**			
Mortality			
30 d	35 (1.3)	63 (2.4)	0.55 (0.33-0.92)
365 d	417 (15.8)	470 (17.8)	0.88 (0.76-1.01)
Readmission			
30 d	457 (17.3)	433 (16.4)	1.06 (0.93-1.20)
365 d	1380 (52.1)	1335 (50.4)	1.05 (0.98-1.13)

Abbreviation: HR, hazard ratio.

### Secondary Outcomes

Patients discharged with diabetes medication intensifications were less likely to die within 30 days of discharge than patients discharged without diabetes medication intensifications (35/2648 [1.3%] vs 63/2648 [2.4%]; HR, 0.55; 95% CI, 0.33-0.92) (Table 2). There was no difference between groups in mortality at 365 days or in all-cause readmissions at 30 or 365 days after discharge.

### Changes in HbA_1c_

**Figure 1.**
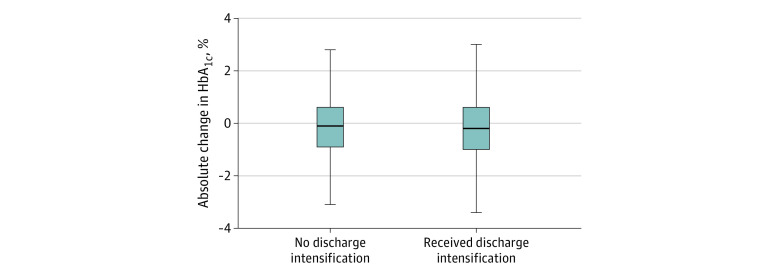
Change in Hemoglobin A_1c_ (HbA_1c_) Values 1 Year After Discharge With or Without Diabetes Medication Intensifications Analysis includes the 4215 patients in the propensity-matched cohort who met the inclusion criteria for the HbA_1c_ analysis (outpatient HbA_1c_ measured between 6 and 18 months after the index hospitalization discharge). Postbaseline HbA_1c_ level was assessed before censoring as the HbA_1c_ level recorded at the closest date to 1 year after index hospitalization discharge, within the range of 6 to 18 months after discharge. Absolute change in HbA_1c_ calculated as postdischarge HbA_1c_ level minus preadmission HbA_1c_ level. Complete difference-in-differences results are given in eTable 4 in the Supplement. The horizontal lines in the center of each box indicate the median; the lower and upper bounds of each box, the 25th and 75th percentiles; the lower and upper error bars, the most extreme value between the 25th percentile minus 1.5 times the interquartile range and the most extreme value between the 75th percentile plus 1.5 times the interquartile range.

Within 1 year of discharge, the mean HbA_1c_ level of patients receiving medication intensifications decreased from 7.91% (95% CI, 7.84%-7.98%) to 7.72% (95% CI, 7.65%-7.79%), and the mean HbA_1c_ level of patients who did not receive intensifications decreased from 7.91% (95% CI, 7.84%-7.97%) to 7.70% (95% CI, 7.68%-7.77%) (Figure 1; eTable 4 in the Supplement). No significant difference was found in the change in HbA_1c_ level between groups (differences-in-differences estimate, 0.02%; 95% CI, −0.12% to 0.16%).

### Persistent Use of Diabetes Medications

**Figure 2.**
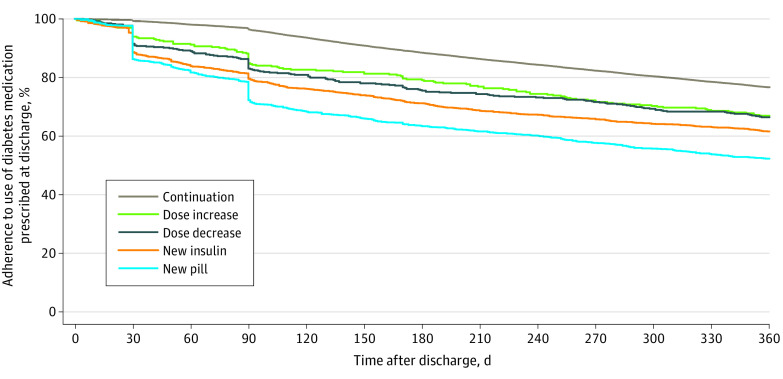
Persistent Use of Diabetes Medications Prescribed at Hospital Discharge by Type of Treatment Persistence analysis includes 18 455 patients who filled 1 or more diabetes medication prescriptions at discharge. A total of 24 085 unique medication prescriptions were included because patients could fill multiple diabetes medication prescriptions at discharge. For each unique medication prescribed at discharge, we calculated persistence as the number of days between the discharge prescription and the last refill for the same or greater dose plus the days supplied by the latest refill. If a patient died during the follow-up period, persistence was truncated at date of death. We reported persistence to 12 months after discharge by type of treatment, and to avoid undercounting because of transient nonadherence, we assessed refill history for 18 months.

In the unmatched cohort, 18 455 patients (65.4%) filled 1 or more diabetes medication prescriptions at discharge and were included in the analyses of postdischarge persistent use of medication. After discharge, 792 of 2298 diabetes medication intensification prescriptions (34.5%) were never filled again, and 378 of 2298 (16.5%) were filled only once after discharge (Figure 2). At 1 year, 48.0% (591 of 1231) of new oral medications and 38.5% (548 of 1423) of new insulin medications were no longer being filled, compared with 23.6% (4858 of 20 550) of same-dose continuations (*P* < .001 for test of difference among medication fill types).

### Prehospitalization Baseline Hemoglobin A_1c_ Subgroup Analyses

Propensity score matching yielded a cohort of 2672 patients with controlled preadmission HbA_1c_ levels (≤7.5%) and a cohort of 2524 patients with elevated preadmission HbA_1c_ levels (>7.5%), each equally split between those who received intensifications and those who did not. Covariate balance between groups in each cohort was excellent except for differences in the regional distribution of patients (eTables 5 and 6 and eFigure 3 in the Supplement).

Among matched patients with controlled baseline HbA_1c_ levels, the mean (SD) prehospitalization HbA_1c_ level was 6.8% (0.5%) for the intensified and not intensified groups. No differences were found in severe hypoglycemia events, severe hyperglycemia events, or secondary clinical outcomes among patients with controlled baseline HbA_1c_ levels who were discharged with or without diabetes medication intensifications (Table 3; eTable 7 in the Supplement).

**Table 3.** Primary Clinical Outcomes Associated With Receiving Diabetes Medication Intensifications at Hospital Discharge in Subgroups With Controlled and Elevated Prehospitalization Hemoglobin A_1c_ Levels

Primary outcome	Controlled (hemoglobin A_1c_ ≤7.5%)			Elevated (hemoglobin A_1c_ >7.5%)		
	Patients, No. (%)		HR (95% CI)	Patients, No. (%)		HR (95% CI)
	Intensified (n = 1336)	Not intensified (n = 1336)		Intensified (n = 1262)	Not intensified (n = 1262)	
Severe hypoglycemia						
30 d	13 (1.0)	11 (0.8)	1.18 (0.55-2.53)	11 (0.9)	8 (0.6)	1.38 (0.51-3.76)
365 d	41 (3.1)	39 (2.9)	1.05 (0.67-1.64)	39 (3.1)	37 (2.9)	1.06 (0.63-1.78)
Severe hyperglycemia						
30 d	4 (0.3)	3 (0.2)	1.34 (0.30-6.00)	4 (0.3)	7 (0.6)	0.57 (0.19-1.72)
365 d	13 (1.0)	7 (0.5)	1.86 (0.74-4.70)	21 (1.7)	27 (2.1)	0.77 (0.44-1.37)

Abbreviation: HR, hazard ratio.

Among matched patients with elevated baseline HbA_1c_ levels, the mean (SD) prehospitalization HbA_1c_ level was 9.1% (1.5%) for the intensified group and 9.1% (1.6%) for the not intensified group. No differences were found in severe hypoglycemia or hyperglycemia events among patients with elevated baseline HbA_1c_ levels who were discharged with or without diabetes medication intensifications (Table 3). Patients who received intensifications were less likely to die within 30 days (HR, 0.46; 95% CI, 0.24-0.91) and 365 days (HR, 0.75; 95% CI, 0.62-0.89) (eTable 7 in the Supplement). Receipt of an intensification was not associated with a significant difference in change in HbA_1c_ level for either subgroup (eTable 8 in the Supplement).

## Discussion

In this cohort study of older adults with diabetes who were hospitalized for common medical conditions, intensification of diabetes medications at hospital discharge was associated with increased short-term risk of severe hypoglycemia events without reduction in risk of severe hyperglycemia events or improvement in HbA_1c_ control at 1 year. Moreover, nearly half of discharge intensifications were not continued at 1 year. Despite the lack of association with improved diabetes control, older adults receiving diabetes medication intensifications at discharge had a lower risk of mortality at 30 days but no difference in mortality at 1 year. These results suggest intensification of older adults’ outpatient diabetes medications during unrelated hospitalizations should generally be avoided.

To our knowledge, no randomized clinical trials to date have evaluated the outcomes of intensifying diabetes medications at hospital discharge. Thus, our findings provide important data to inpatient clinicians considering discharge changes to home diabetes medications. Two prior observational studies^6,39^ examined outcomes of patients discharged with diabetes medication intensifications. A single-center study^39^ conducted from 2007 to 2009 found no overall difference in 30-day readmissions among adults receiving intensifications but did not examine hypoglycemia or hyperglycemia events and was unable to examine readmissions to other hospitals. A cohort study^6^ of older adults with diabetes hospitalized in Ontario, Canada, between 2004 and 2013 found that discharge with new insulin was associated with an increased risk of death and readmissions compared with using oral diabetes medications. This study differed from our analysis by including patients hospitalized for diabetes and surgical conditions as well as medical hospitalizations. This study also lacked key covariates, including vital signs and laboratory values, such as HbA_1c_, which may confound the association between discharge intensification and discharge outcomes.^6^ Our study builds on these prior studies^6,39^ by examining postdischarge outcomes in the VHA, a large, national, integrated health system, which allows for the inclusion of richer clinical characteristics and complete identification of postdischarge events that occur inside and outside the VHA. In addition, we examined a more recent period than prior studies^6,39^; thus, differences in findings may in part reflect differences in diabetes medication classes.

In a secondary analysis, we observed that older adults receiving diabetes intensifications had a substantially lower risk of death in the first 30 days of discharge. This unexpected finding was consistent in elevated but not controlled HbA_1c_ subgroups and merits further examination in future studies. This finding contrasts with the Canadian study,^6^ which found that discharge with new insulin was associated with an increased risk of death, and a prior trial^40^ of intensive inpatient blood glucose control among critically ill patients, which found more intensive blood glucose control was associated with higher 90-day mortality. We anticipate this finding may be attributable to unmeasured cofounding because we did not observe a concomitant lower risk of readmissions or serious hyperglycemia events. Randomized clinical trials of intensive diabetes treatment have not clearly established a mortality benefit and have typically studied younger and healthier populations.^30^ Furthermore, time to benefit from intensive diabetes treatment is typically measured in years^41^; thus, it is unlikely that a large mortality benefit is the result of a short-term decision about chronic disease treatment. Instead, clinicians may be appropriately identifying certain patients at high short-term risk of death and choosing not to intensify their diabetes medication regimens based on factors not captured by our propensity score. Although we were able to include markers of comorbidity, frailty, vital signs, laboratory values, and medications in the propensity score and excluded patients discharged to hospice or skilled nursing facilities, identifying comorbidities based on diagnosis coding does not account for differences in disease severity (eg, well-controlled vs end-stage heart failure) or functional status. Some patients may have been offered hospice or skilled nursing facility care and declined these services. Of importance, because our primary survival analyses models accounted for competing risks of death, this unanticipated finding should not bias the primary outcomes.

Most older adults discharged with intensified diabetes medications in this study received new insulin or sulfonylureas, which carry a higher risk of hypoglycemia than other diabetes medication classes. Novel classes, such as SGLT2 inhibitors and GLP-1 agonists, may have different benefit-harm profiles owing to lower hypoglycemia risks and strong cardioprotective benefits; thus, our study findings, which examined a period before the widespread use of these new classes, do not extend to these classes. Dedicated studies on the real-world outcomes of initiation of use of SGLT2 inhibitors and GLP-1 agonists among hospitalized older adults are crucial before recommending their routine use in this clinical setting because the clinical trials participants in which these classes have been studied are likely to be younger and healthier than hospitalized populations and thus the safety profile and benefits of these classes may differ in the acutely ill population. As the current study and a prior study^30^ of antihypertensives intensifications suggest, medications intensified at hospital discharge are frequently discontinued, thus exposing patients to short-term risks without a chance for long-term benefits.

How then should inpatient clinicians manage older adults’ diabetes medications at hospital discharge? For most older adults with well-controlled or modestly elevated HbA_1c_ levels, deferring decisions to intensify treatment to outpatient clinicians is likely the safest course because this practice avoids the tendency to treat elevated inpatient blood glucose values, which are typically transitory, with a change to long-term therapy that may result in increased risk of severe hypoglycemia. Older adults with uncontrolled diabetes warrant close follow up with their outpatient clinicians, but in most cases treatment intensifications may still be deferred because, as our study suggests, readmission for hyperglycemia is rare, occurring in less than 0.5% of cohort patients with elevated baseline HbA_1c_ levels. Adverse drug events are typically highest in the initial weeks of treatment, and this risk is likely to be multiplied in the postdischarge period, during which older adults are typically exposed to multiple medication changes and hospital-associated disability.^20,21,42^ Additional research is needed to determine best management practices for older adults with diabetes whose primary reason for hospitalization requires short-term treatment with medications known to greatly increase blood glucose levels, which will be continued to the outpatient setting (eg, corticosteroids for respiratory or autoimmune disease flairs) because these patients may benefit from short-term monitored intensifications.

### Limitations

Our study has several limitations. First, it took place in the VHA health care system, a national integrated system that serves a predominately male population with greater multimorbidity that may not be generalizable to the entire US. Second, because we focused on older adults who are at higher risk of adverse drug events, our findings are not generalizable to younger populations. Third, although our study was strengthened by examining both VHA and Medicare data, we were not able to identify hyperglycemia and hypoglycemia events for which patients did not seek emergency care; thus, these events are likely underestimated.^43^ Fourth, because of limitations of pharmacy data, we were unable to examine the impact of changes in insulin dosing and thus did not examine patients taking insulin before hospitalization. Pharmacy claims do not allow for the identification of discontinued medications; thus, medication classes started as substitutions for other classes were included in the study as intensifications. Fifth, as an observational study, there is a risk of unmeasured confounding by variables not included in the propensity score–matched analyses. Sixth, subgroup analyses were exploratory and may have been underpowered to demonstrate differential associations of diabetes medication intensification across baseline HbA_1c_ levels.

## Conclusions

Among older adults hospitalized for common medical conditions, discharge with intensified diabetes medications was not associated with reduced severe hyperglycemia events or HbA_1c_ levels within 1 year but was associated with an increased risk of severe hypoglycemia events within 30 days. For most patients with elevated inpatient blood glucose levels, communication of concerns about patients’ diabetes control to patients and their outpatient clinicians for close follow-up may provide a safer path than intensifying diabetes medications at discharge.

## Notes

Supplement. **eTable 1.** Detailed Outcome Definitions **eTable 2.** Diabetes Medication Intensifications at Hospital Discharge **eTable 3.** Complete Cohort Characteristics Before and After Propensity Score Matching **eTable 4.** Difference in Differences Analysis of Hemoglobin A_1c_ Values One Year Following Discharge With or Without Diabetes Medication Intensifications **eTable 5.** Baseline Characteristics Before and After Propensity Score Matching, Controlled Baseline HbA_1c_ Subgroup **eTable 6.** Baseline Characteristics Before and After Propensity Score Matching, Elevated Baseline HbA_1c_ Subgroup **eTable 7.** Secondary Clinical Outcomes Associated With Receiving Diabetes Medication Intensifications at Hospital Discharge in Subgroups With Controlled and Elevated Prehospitalization Hemoglobin A_1c_ Subgroups **eTable 8.** Difference in Differences Analysis of Hemoglobin A_1c_ Values One Year Following Discharge With or Without Diabetes Medication Intensifications in Controlled and Elevated Baseline HbA_1c_ Subgroups **eFigure 1.** Flow Diagram of Underlying Study Population **eFigure 2.** Propensity Score Distributions Before and After Matching **eFigure 3.** Propensity Score Distributions Before and After Matching in Controlled and Elevated Baseline HbA_1c_ Subgroups

## References

[1] Unroe KT, Pfeiffenberger T, Riegelhaupt S, Jastrzembski J, Lokhnygina Y, Colón-Emeric C. Inpatient medication reconciliation at admission and discharge: a retrospective cohort study of age and other risk factors for medication discrepancies. Am J Geriatr Pharmacother. 2010;8(2):115-126. doi:10.1016/j.amjopharm.2010.04.002 20439061 PMC3740385

[2] Harris CM, Sridharan A, Landis R, Howell E, Wright S. What happens to the medication regimens of older adults during and after an acute hospitalization? J Patient Saf. 2013;9(3):150-153. doi:10.1097/PTS.0b013e318286f87d 23965837

[3] Bell CM, Brener SS, Gunraj N, . Association of ICU or hospital admission with unintentional discontinuation of medications for chronic diseases. JAMA. 2011;306(8):840-847. doi:10.1001/jama.2011.1206 21862745

[4] Anderson TS, Wray CM, Jing B, . Intensification of older adults’ outpatient blood pressure treatment at hospital discharge: national retrospective cohort study. BMJ. 2018;362:k3503. doi:10.1136/bmj.k3503 30209052 PMC6283373

[5] Anderson TS, Lee S, Jing B, . Prevalence of diabetes medication intensifications in older adults discharged from US Veterans Health Administration hospitals. JAMA Netw Open. 2020;3(3):e201511. doi:10.1001/jamanetworkopen.2020.1511 32207832 PMC7093767

[6] Lysy Z, Fung K, Giannakeas V, Fischer HD, Bell CM, Lipscombe LL. The association between insulin initiation and adverse outcomes after hospital discharge in older adults: a population-based cohort study. J Gen Intern Med. 2019;34(4):575-582. doi:10.1007/s11606-019-04849-3 30756304 PMC6445910

[7] Umpierrez GE, Isaacs SD, Bazargan N, You X, Thaler LM, Kitabchi AE. Hyperglycemia: an independent marker of in-hospital mortality in patients with undiagnosed diabetes. J Clin Endocrinol Metab. 2002;87(3):978-982. doi:10.1210/jcem.87.3.8341 11889147

[8] Turchin A, Matheny ME, Shubina M, Scanlon JV, Greenwood B, Pendergrass ML. Hypoglycemia and clinical outcomes in patients with diabetes hospitalized in the general ward. Diabetes Care. 2009;32(7):1153-1157. doi:10.2337/dc08-2127 19564471 PMC2699723

[9] Garg R, Hurwitz S, Turchin A, Trivedi A. Hypoglycemia, with or without insulin therapy, is associated with increased mortality among hospitalized patients. Diabetes Care. 2013;36(5):1107-1110. doi:10.2337/dc12-1296 23248192 PMC3631882

[10] Sahni N, Simon G, Arora R. Finding the sweet spot: the last blood glucose measured in the hospital and 30-day outcomes: a retrospective study. J Gen Intern Med. 2019;34(4):510-512. doi:10.1007/s11606-018-4740-z 30552601 PMC6446001

[11] Driver BE, Olives TD, Bischof JE, Salmen MR, Miner JR. Discharge glucose is not associated with short-term adverse outcomes in emergency department patients with moderate to severe hyperglycemia. Ann Emerg Med. 2016;68(6):697-705.e3. doi:10.1016/j.annemergmed.2016.04.057 27353284

[12] Inzucchi SE. Clinical practice: management of hyperglycemia in the hospital setting. N Engl J Med. 2006;355(18):1903-1911. doi:10.1056/NEJMcp060094 17079764

[13] Umpierrez GE, Hellman R, Korytkowski MT, ; Endocrine Society. Management of hyperglycemia in hospitalized patients in non-critical care setting: an endocrine society clinical practice guideline. J Clin Endocrinol Metab. 2012;97(1):16-38. doi:10.1210/jc.2011-2098 22223765

[14] American Diabetes Association. Diabetes care in the hospital: standards of medical care in diabetes-2019*.* Diabetes Care. 2019;42(suppl 1):S173-S181. doi:10.2337/dc19-S015 30559241

[15] Lipska KJ, Ross JS, Wang Y, . National trends in US hospital admissions for hyperglycemia and hypoglycemia among Medicare beneficiaries, 1999 to 2011. JAMA Intern Med. 2014;174(7):1116-1124. doi:10.1001/jamainternmed.2014.1824 24838229 PMC4152370

[16] McCoy RG, Van Houten HK, Ziegenfuss JY, Shah ND, Wermers RA, Smith SA. Increased mortality of patients with diabetes reporting severe hypoglycemia. Diabetes Care. 2012;35(9):1897-1901. doi:10.2337/dc11-2054 22699297 PMC3425008

[17] Geller AI, Shehab N, Lovegrove MC, . National estimates of insulin-related hypoglycemia and errors leading to emergency department visits and hospitalizations. JAMA Intern Med. 2014;174(5):678-686. doi:10.1001/jamainternmed.2014.136 24615164 PMC4631022

[18] Lipska KJ, Krumholz H, Soones T, Lee SJ. Polypharmacy in the aging patient: a review of glycemic control in older adults with type 2 diabetes. JAMA. 2016;315(10):1034-1045. doi:10.1001/jama.2016.0299 26954412 PMC4823136

[19] American Diabetes Association. Older adults: standards of medical care in diabetes-2020*.* Diabetes Care. 2020;43(suppl 1):S152-S162. doi:10.2337/dc20-S012 31862755

[20] Forster AJ, Murff HJ, Peterson JF, Gandhi TK, Bates DW. The incidence and severity of adverse events affecting patients after discharge from the hospital. Ann Intern Med. 2003;138(3):161-167. doi:10.7326/0003-4819-138-3-200302040-00007 12558354

[21] Krumholz HM. Post-hospital syndrome: an acquired, transient condition of generalized risk. N Engl J Med. 2013;368(2):100-102. doi:10.1056/NEJMp1212324 23301730 PMC3688067

[22] Lee AK, Steinman MA, Lee SJ. Improving the American Diabetes Association Framework for individualizing treatment in older adults: evaluating life expectancy. BMJ Open Diabetes Res Care. 2020;8(1):e001624. doi:10.1136/bmjdrc-2020-001624 32988850 PMC7523213

[23] Anderson TS, Odden M, Penko J, Kazi DS, Bellows BK, Bibbins-Domingo K. Generalizability of clinical trials supporting the 2017 American College of Cardiology/American Heart Association blood pressure guideline. JAMA Intern Med. 2020;180(5):795-797. doi:10.1001/jamainternmed.2020.0051 32176252 PMC7076539

[24] Kerr EA, Lucatorto MA, Holleman R, Hogan MM, Klamerus ML, Hofer TP; VA Diabetes Quality Enhancement Research Initiative (QUERI) Workgroup on Clinical Action Measures. Monitoring performance for blood pressure management among patients with diabetes mellitus: too much of a good thing? Arch Intern Med. 2012;172(12):938-945. doi:10.1001/archinternmed.2012.2253 22641246 PMC3699173

[25] Hebert PL, Geiss LS, Tierney EF, Engelgau MM, Yawn BP, McBean AM. Identifying persons with diabetes using Medicare claims data. Am J Med Qual. 1999;14(6):270-277. doi:10.1177/106286069901400607 10624032

[26] Anderson TS, Jing B, Wray CM, . Comparison of pharmacy database methods for determining prevalent chronic medication use. Med Care. 2019;57(10):836-842. doi:10.1097/MLR.0000000000001188 31464843 PMC6742560

[27] Anderson TS, Xu E, Whitaker E, Steinman MA. A systematic review of methods for determining cross-sectional active medications using pharmacy databases. Pharmacoepidemiol Drug Saf. 2019;28(4):403-421. doi:10.1002/pds.4706 30761662 PMC7050409

[28] Ginde AA, Blanc PG, Lieberman RM, Camargo CA Jr. Validation of ICD-9-CM coding algorithm for improved identification of hypoglycemia visits. BMC Endocr Disord. 2008;8:4. doi:10.1186/1472-6823-8-4 18380903 PMC2323001

[29] Karter AJ, Warton EM, Moffet HH, . Revalidation of the hypoglycemia risk stratification tool Using ICD-10 codes. Diabetes Care. 2019;42(4):e58-e59. doi:10.2337/dc18-2154 30765427 PMC6429629

[30] Gerstein HC, Miller ME, Byington RP, ; Action to Control Cardiovascular Risk in Diabetes Study Group. Effects of intensive glucose lowering in type 2 diabetes. N Engl J Med. 2008;358(24):2545-2559. doi:10.1056/NEJMoa0802743 18539917 PMC4551392

[31] Dharmarajan K, Hsieh AF, Lin Z, . Diagnoses and timing of 30-day readmissions after hospitalization for heart failure, acute myocardial infarction, or pneumonia. JAMA. 2013;309(4):355-363. doi:10.1001/jama.2012.216476 23340637 PMC3688083

[32] Austin PC. An introduction to propensity score methods for reducing the effects of confounding in observational studies. Multivariate Behav Res. 2011;46(3):399-424. doi:10.1080/00273171.2011.568786 21818162 PMC3144483

[33] Austin PC. Balance diagnostics for comparing the distribution of baseline covariates between treatment groups in propensity-score matched samples. Stat Med. 2009;28(25):3083-3107. doi:10.1002/sim.3697 19757444 PMC3472075

[34] Austin PC, Lee DS, Fine JP. Introduction to the analysis of survival data in the presence of competing risks. Circulation. 2016;133(6):601-609. doi:10.1161/CIRCULATIONAHA.115.017719 26858290 PMC4741409

[35] Meyer BD. Natural and quasi-experiments in economics. J Bus Econ Stat. 1995;13(2):151-161. doi:10.2307/1392369

[36] Lipska KJ, Parker MM, Moffet HH, Huang ES, Karter AJ. Association of initiation of basal insulin analogs vs neutral protamine hagedorn insulin with hypoglycemia-related emergency department visits or hospital admissions and with glycemic control in patients with type 2 diabetes. JAMA. 2018;320(1):53-62. doi:10.1001/jama.2018.7993 29936529 PMC6134432

[37] Raebel MA, Schmittdiel J, Karter AJ, Konieczny JL, Steiner JF. Standardizing terminology and definitions of medication adherence and persistence in research employing electronic databases. Med Care. 2013;51(8)(suppl 3):S11-S21. doi:10.1097/MLR.0b013e31829b1d2a 23774515 PMC3727405

[38] Vasilevskis EE. The challenges of diabetes medication management at hospital discharge in older adults. JAMA Netw Open. 2020;3(3):e201500. doi:10.1001/jamanetworkopen.2020.1500 32207829

[39] Wei NJ, Wexler DJ, Nathan DM, Grant RW. Intensification of diabetes medication and risk for 30-day readmission. Diabet Med. 2013;30(2):e56-e62. doi:10.1111/dme.12061 23126686 PMC3552066

[40] Finfer S, Chittock DR, Su SY, ; NICE-SUGAR Study Investigators. Intensive versus conventional glucose control in critically ill patients. N Engl J Med. 2009;360(13):1283-1297. doi:10.1056/NEJMoa0810625 19318384

[41] Lee SJ, Leipzig RM, Walter LC. Incorporating lag time to benefit into prevention decisions for older adults. JAMA. 2013;310(24):2609-2610. doi:10.1001/jama.2013.282612 24322396 PMC4049260

[42] Karter AJ, Moffet HH, Liu JY, Lipska KJ. Surveillance of hypoglycemia—limitations of emergency department and hospital utilization data. JAMA Intern Med. 2018;178(7):987-988. doi:10.1001/jamainternmed.2018.1014 29710182 PMC6033620

[43] Anderson TS, Jing B, Fung K, Steinman MA. Older adults’ persistence to antihypertensives prescribed at hospital discharge: a retrospective cohort study. J Gen Intern Med. 2021. doi:10.1007/s11606-020-06401-0 33469765 PMC8642581

