# Supplementary material for: Intensification of Diabetes Medications at Hospital Discharge and Clinical Outcomes in Older Adults in the Veterans Administration Health System
Source: JAMA Netw Open. 2021 Oct 21;4(10):e2128998. doi: 10.1001/jamanetworkopen.2021.28998 (PMC8531994; doi:10.1001/jamanetworkopen.2021.28998)
Supplement: Supplement. — eTable 1. Detailed Outcome Definitions eTable 2. Diabetes Medication Intensifications at Hospital Discharge eTable 3. Complete Cohort Characteristics Before and After Propensity Score Matching eTable 4. Difference in Differences Analysis of Hemoglobin A1c Values One Year Following Discharge With or Without Diabetes Medication Intensifications eTable 5. Baseline Characteristics Before and After Propensity Score Matching, Controlled Baseline HbA1c Subgroup eTable 6. Baseline Characteristics Before and After Propensity Score Matching, Elevated Baseline HbA1c Subgroup eTable 7. Secondary Clinical Outcomes Associated With Receiving Diabetes Medication Intensifications at Hospital Discharge in Subgroups With Controlled and Elevated Prehospitalization Hemoglobin A1c Subgroups eTable 8. Difference in Differences Analysis of Hemoglobin A1c Values One Year Following Discharge With or Without Diabetes Medication Intensifications in Controlled and Elevated Baseline HbA1c Subgroups eFigure 1. Flow Diagram of Underlying Study Population eFigure 2. Propensity Score Distributions Before and After Matching eFigure 3. Propensity Score Distributions Before and After Matching in Controlled and Elevated Baseline HbA1c Subgroups [file jamanetwopen-e2128998-s001.pdf]

Supplementary Online Content

Anderson TS, Lee AK, Jing B, et al. Intensification of diabetes medications at hospital discharge and clinical outcomes in older adults in the Veterans Administration health system. *JAMA Netw Open*. 2021;4(10):e2128998. doi:10.1001/jamanetworkopen.2021.28998

eTable 1. Detailed Outcome Definitions

eTable 2. Diabetes Medication Intensifications at Hospital Discharge

eTable 3. Complete Cohort Characteristics Before and After Propensity Score Matching

eTable 4. Difference in Differences Analysis of Hemoglobin A_{1c} Values One Year Following Discharge With or Without Diabetes Medication Intensifications

eTable 5. Baseline Characteristics Before and After Propensity Score Matching, Controlled Baseline HbA_{1c} Subgroup

eTable 6. Baseline Characteristics Before and After Propensity Score Matching, Elevated Baseline HbA_{1c} Subgroup

eTable 7. Secondary Clinical Outcomes Associated With Receiving Diabetes Medication Intensifications at Hospital Discharge in Subgroups With Controlled and Elevated Prehospitalization Hemoglobin A_{1c} Subgroups

eTable 8. Difference in Difference Analysis of Hemoglobin A_{1c} Values One Year Following Discharge With or Without Diabetes Medication Intensifications in Controlled and Elevated Baseline HbA_{1c} Subgroups

eFigure 1. Flow Diagram of Underlying Study Population

eFigure 2. Propensity Score Distributions Before and After Matching

eFigure 3. Propensity Score Distributions Before and After Matching in Controlled and Elevated Baseline HbA_{1c} Subgroups

This supplementary material has been provided by the authors to give readers additional information about their work.

eTable 1. Outcome definitions

Outcome	Setting	ICD-9 Codes	ICD-10 Codes
Severe hypoglycemia	Medicare or VA emergency department, observation stay or inpatient hospitalization claim with a primary discharge diagnosis code of:	251.0, 251.1, 251.2, 962.3<br><br>250.8, without concurrent 259.8, 272.7, 681.XX, 682.XX, 686.9X, 707.1-707.9, 709.3, 730.0-730.2, or 731.8 codes	E08.641, E08.649, E09.641, E09.649, E10.641, E10.649, E11.641, E11.649, E13.641, E13.649, E15, E16.0, E16.1, E16.2, T38.3X1A, T38.3X1D,T38.3X1S, T38.3X2A, T38.3X2D, T38.3X2S, T38.3X3A, T38.3X3D, T38.3X3S, T38.3X4A, T38.3X4D, T38.3X4S, T38.3X5A, T38.3X5D, T38.3X5S
Severe hyperglycemia	Medicare or VA emergency department, observation stay or inpatient hospitalization claim with a primary discharge diagnosis code of:	250.02, 250.03, 250.1X, 250.2X, 250.3X	E0800, E0801, E0810, E0811, E0865<br>E0900, E0901, E0910, E0911, E0965<br>E1000, E1001, E1010, E1011, E1065<br>E1100, E1101, E1110, E1111, E1165<br>E1300, E1301, E1310, E1311, E1365

Sources:

Ginde AA, Blanc PG, Lieberman RM, Camargo CA Jr. Validation of ICD-9-CM coding algorithm for improved identification of hypoglycemia visits. *BMC Endocr Disord*. 2008;8:4.

Lipska KJ, Ross JS, Wang Y, Inzucchi SE, Minges K, Karter AJ, Huang ES, Desai MM, Gill TM, Krumholz HM. National trends in US hospital admissions for hyperglycemia and hypoglycemia among Medicare beneficiaries, 1999 to 2011. *JAMA Intern Med*. 2014 Jul;174(7):1116-24.

Karter AJ, Warton EM, Moffet HH, Ralston JD, Huang ES, Miller DR, Lipska KJ. Revalidation of the Hypoglycemia Risk Stratification Tool Using ICD-10 Codes. *Diabetes Care*. 2019 Apr;42(4):e58-e59.

© 2021 Anderson TS et al. *JAMA Network Open.*

eTable 2. Diabetes medication intensifications at hospital discharge

Intensification type, No. (%)	Overall (N=28,198)	Preadmission HbA_{1c} ≤7.5% (N=20,267)	Preadmission HbA_{1c} >7.5% (N=7,061)
Any intensification	2768 (9.8)	1341 (6.6)	1350 (19.1)
**New medication starts [a]**			
New insulin	1423 (5.1)	542 (2.7)	838 (11.9)
New long-acting insulin	1213 (4.3)	413 (2.0)	761 (10.8)
New short-acting insulin	593 (2.1)	238 (1.2)	340 (4.8)
New sulfonylurea	630 (2.2)	365 (1.8)	249 (3.5)
New biguanide	522 (1.9)	278 (1.4)	234 (3.3)
New DPP4 inhibitor	45 (0.2)	17 (0.1)	28 (0.4)
New alpha-glucosidase inhibitor	15 (0.1)	8 (<0.1)	5 (0.1)
New meglitinide	9 (<0.1)	4 (<0.1)	4 (0.1)
New thiazolidinedione	8 (<0.1)	4 (<0.1)	4 (0.1)
New GLP1 receptor agonists	1 (<0.1)	1 (<0.1)	0 (0.0)
New SGLT2 inhibitor	0 (0.0)	0 (0.0)	0 (0.0)
**Dose increases [b]**			
Increased sulfonylurea	210 (0.7)	114 (0.6)	88 (1.3)
Increased biguanide	151 (0.5)	94 (0.5)	51 (0.7)
Increased DPP4 inhibitor	0 (0.0)	0 (0.0)	0 (0.0)
Increased alpha-glucosidase inhibitor	3 (<0.1)	1 (<0.1)	2 (<0.1)
Increased meglitinide	0 (0.0)	0 (0.0)	0 (0.0)
Increased thiazolidinedione	0 (0.0)	0 (0.0)	0 (0.0)
Increased GLP1 receptor agonist	0 (0.0)	0 (0.0)	0 (0.0)
Increased SGLT2 inhibitor	0 (0.0)	0 (0.0)	0 (0.0)

Abbreviations: HbA_{1c}, hemoglobin A1c; GLP-1, glucagon-like peptide-1; DPP4, dipeptidyl peptidase 4; SGLT2, sodium glucose co-transporter 2 inhibitor

[a] Medication start defined as discharge prescription for medication class not used on admission.
[b] Dose increases defined as >20% increase in total daily dose of home medication class being used prior to hospitalization.

© 2021 Anderson TS et al. *JAMA Network Open.*

eTable 3. Complete Cohort Characteristics Before and After Propensity Score Matching

	Before Propensity Score Matching			After Propensity Score Matching		
**Characteristic [a]**	**Intensified (N= 2,768)**	**Not Intensified (N= 25,430)**	**SMD**	**Intensified (N= 2,648)**	**Not Intensified (N= 2,648)**	**SMD**
Age, mean (SD), y	72.6 (7.3)	73.8 (7.7)	0.16	72.7 (7.3)	72.8 (7.3)	0.01
Male sex, No. (%)	2721 (98.3)	24989 (98.3)	0.00	2603 (98.3)	2609 (98.5)	0.02
Race/ethnicity, No. (%)			0.09			0.02
White	2155 (77.9)	20445 (80.4)		2074 (78.3)	2064 (77.9)	
Black	456 (16.5)	3704 (14.6)		426 (16.1)	441 (16.7)	
Hispanic	28 (1.0)	366 (1.4)		26 (1.0)	21 (0.8)	
Other	129 (4.7)	915 (3.6)		122 (4.6)	122 (4.6)	
Median zip code household income, mean (SD), $	26571.5 (44152.3)	27167.2 (45854.5)	0.01	26645.3 (44848.4)	27717.4 (49144.8)	0.02
VISN, No. (%)			0.18			0.07
New England Health Care System	102 (3.7)	1215 (4.8)		100 (3.8)	106 (4.0)	
Healthcare New York/New Jersey	158 (5.7)	1283 (5.0)		152 (5.7)	150 (5.7)	
VISN4 Network	125 (4.5)	1141 (4.5)		119 (4.5)	113 (4.3)	
Capitol Healthcare Network	84 (3.0)	659 (2.6)		79 (3.0)	95 (3.6)	
Mid-Atlantic Health Care Network	156 (5.6)	1555 (6.1)		151 (5.7)	152 (5.7)	
Southeast Network	179 (6.5)	1171 (4.6)		166 (6.3)	174 (6.6)	
Sunshine Healthcare Network	212 (7.7)	2488 (9.8)		206 (7.8)	192 (7.3)	
MidSouth Healthcare Network	156 (5.6)	1856 (7.3)		152 (5.7)	157 (5.9)	
VISN 10 Healthcare System	104 (3.8)	858 (3.4)		100 (3.8)	102 (3.9)	
Great Lakes Healthcare System	278 (10.0)	2329 (9.2)		256 (9.7)	260 (9.8)	
Heartland Network	129 (4.7)	1275 (5.0)		127 (4.8)	128 (4.8)	
South Central VA Healthcare Network	342 (12.4)	2551 (10.0)		330 (12.5)	336 (12.7)	
Heart of Texas Healthcare System	104 (3.8)	1313 (5.2)		100 (3.8)	87 (3.3)	
Southwest Healthcare Network	95 (3.4)	956 (3.8)		88 (3.3)	73 (2.8)	
Rocky Mountain Network	72 (2.6)	608 (2.4)		69 (2.6)	67 (2.5)	
Northwest Health Network	89 (3.2)	889 (3.5)		86 (3.2)	99 (3.7)	
Sierra Pacific Network	103 (3.7)	1020 (4.0)		99 (3.7)	107 (4.0)	
Desert Pacific Healthcare Network	164 (5.9)	1338 (5.3)		153 (5.8)	138 (5.2)	
Midwest Healthcare Network	116 (4.2)	925 (3.6)		115 (4.3)	112 (4.2)	
**Pre-admission clinical characteristics, mean (SD)**						
Body mass index, kg/m^2	31.2 (6.6)	30.8 (6.5)	0.06	31.2 (6.5)	31.0 (6.7)	0.03
Systolic BP, mmHg	134.8 (17.9)	133.0 (17.5)	0.10	134.7 (17.9)	134.7 (17.9)	0.00
Diastolic BP, mmHg	73.5 (10.4)	72.4 (10.2)	0.10	73.4 (10.4)	73.3 (10.5)	0.01

© 2021 Anderson TS et al. *JAMA Network Open.*

Frailty score	0.2 (0.1)	0.2 (0.1)	0.02	0.2 (0.1)	0.2 (0.1)	0.01
**Pre-admission diabetes management**						
Hemoglobin A1c, mean (SD), d	8.0 (1.7)	7.1 (1.1)	0.68	7.9 (1.5)	7.9 (1.7)	0.02
Estimated glomerular filtration rate, mean (SD), mL/min per 1.73 m2	65.5 (23.8)	67 (24.3)	0.06	65.5 (23.7)	65.5 (24.0)	0.00
Any hypoglycemia hospitalizations in prior year, No. (%)	38 (1.4)	211 (0.8)	0.05	36 (1.4)	53 (2.0)	0.05
Number of admission diabetes medications, No. (%)			0.31			0.04
0	1062 (38.4)	6311 (24.8)		974 (36.8)	988 (37.3)	
1	1073 (38.8)	13113 (51.6)		1046 (39.5)	1003 (37.9)	
2	547 (19.8)	5349 (21.0)		542 (20.5)	559 (21.1)	
>3	86 (3.2)	657 (2.6)		86 (3.3)	98 (3.7)	
Admission diabetes medication classes, No. (%) [b]						
Metformin	1071 (38.7)	12462 (49.0)	0.21	1056 (39.9)	1038 (39.2)	0.01
Sulfonylureas	1127 (40.7)	11758 (46.2)	0.11	1108 (41.8)	1140 (43.1)	0.02
Thiazolidinediones	107 (3.9)	696 (2.7)	0.06	105 (4.0)	108 (4.1)	0.01
α-glucosidase inhibitors	66 (2.4)	373 (1.5)	0.07	66 (2.5)	71 (2.7)	0.01
Dipeptidyl peptidase-4 inhibitors	45 (1.6)	357 (1.4)	0.02	44 (1.7)	53 (2.0)	0.03
Other classes	4 (0.1)	50 (0.2)	0.01	4 (0.2)	2 (0.1)	0.02
Preadmission diabetes medication adherence			0.31			0.02
0 medications	1062 (38.4)	6311 (24.8)		974 (36.8)	988 (37.3)	
PDC ≤ 80%	616 (22.3)	5805 (22.8)		604 (22.8)	613 (23.1)	
PDC > 80%	1090 (39.4)	13314 (52.4)		1070 (40.4)	1047 (39.5)	
Diabetes medication classes used in prior year, No. (%)						
Metformin	1448 (52.3)	13724 (54.0)	0.03	1383 (52.2)	1370 (51.7)	0.01
Sulfonylureas	1482 (53.5)	12759 (50.2)	0.07	1427 (53.9)	1458 (55.1)	0.02
Thiazolidinediones	140 (5.1)	863 (3.4)	0.08	137 (5.2)	143 (5.4)	0.01
α-glucosidase inhibitors	88 (3.2)	454 (1.8)	0.09	87 (3.3)	90 (3.4)	0.01
Dipeptidyl peptidase-4 inhibitors	35 (1.3)	246 (1.0)	0.03	32 (1.2)	46 (1.7)	0.04
Insulins	645 (23.3)	1370 (5.4)	0.53	552 (20.8)	570 (21.5)	0.02
Other classes	9 (0.3)	79 (0.3)	0.00	9 (0.3)	6 (0.2)	0.02
**Pre-admission healthcare use, No. (%)**						
Any Medicare utilization in prior year, No. (%)	818 (29.6)	7722 (30.4)	0.02	783 (29.6)	796 (30.1)	0.01
No. ED visits in the year preceding index hospitalization			0.07			0.07
0	1418 (51.2)	12746 (50.1)		1364 (51.5)	1373 (51.9)	
1	676 (24.4)	6063 (23.8)		650 (24.5)	629 (23.8)	
2	349 (12.6)	3084 (12.1)		326 (12.3)	334 (12.6)	
≥3	325 (11.7)	3537 (13.9)		308 (11.6)	312 (11.8)	

© 2021 Anderson TS et al. *JAMA Network Open.*

No. hospitalizations in the year preceding index hospitalization			0.02			0.01
0	1957 (70.7)	18077 (71.1)		1885 (71.2)	1887 (71.3)	
1	480 (17.3)	4488 (17.6)		450 (17.0)	457 (17.3)	
2	186 (6.7)	1648 (6.5)		175 (6.6)	169 (6.4)	
≥3	145 (5.2)	1217 (4.8)		138 (5.2)	135 (5.1)	
Admission medication count, mean (SD)	7.9 (4.7)	8.9 (4.9)	0.22	8 (4.7)	8 (5.0)	0.00
Polypharmacy (>8 medications), No. (%)	1434 (51.8)	15608 (61.4)	0.19	1402 (52.9)	1410 (53.2)	0.01
**Index hospitalization characteristics**						
Length of stay, mean (SD), d	6.6 (8.0)	5.2 (5.8)	0.20	6.5 (8.1)	6.4 (8.7)	0.01
Year of hospitalization, No. (%)			0.06			0.04
2011	646 (23.3)	5714 (22.5)		619 (23.4)	624 (23.6)	
2012	496 (17.9)	4551 (17.9)		477 (18.0)	481 (18.2)	
2013	460 (16.6)	4237 (16.7)		443 (16.7)	422 (15.9)	
2014	447 (16.1)	4058 (16.0)		423 (16.0)	411 (15.5)	
2015	380 (13.7)	3953 (15.5)		363 (13.7)	392 (14.8)	
2016	339 (12.2)	2917 (11.5)		323 (12.2)	318 (12.0)	
Training hospital, No. (%)	2451 (88.5)	22790 (89.6)	0.03	2352 (88.8)	2381 (89.9)	0.04
**Discharge diagnoses, No. (%)**			0.18			0.08
Arrhythmia	18 (0.7)	370 (1.5)		18 (0.7)	17 (0.6)	
Asthma	22 (0.8)	124 (0.5)		21 (0.8)	19 (0.7)	
Chronic obstructive pulmonary disease	267 (9.6)	2156 (8.5)		255 (9.6)	264 (10.0)	
Chest pain	82 (3.0)	940 (3.7)		80 (3.0)	72 (2.7)	
Conduction disorders	212 (7.7)	2737 (10.8)		206 (7.8)	178 (6.7)	
Coronary artery disease	356 (12.9)	2794 (11)		341 (12.9)	356 (13.4)	
Acute coronary syndrome	172 (6.2)	1520 (6.0)		162 (6.1)	175 (6.6)	
Heart failure	484 (17.5)	3854 (15.2)		464 (17.5)	482 (18.2)	
Heart valve disorders	65 (2.3)	588 (2.3)		65 (2.5)	62 (2.3)	
Pneumonia	275 (9.9)	2830 (11.1)		268 (10.1)	255 (9.6)	
Sepsis	77 (2.8)	797 (3.1)		68 (2.6)	59 (2.2)	
Skin infection	246 (8.9)	2261 (8.9)		231 (8.7)	223 (8.4)	
Stroke	175 (6.3)	1320 (5.2)		164 (6.2)	197 (7.4)	
Transient ischemic attack	37 (1.3)	415 (1.6)		35 (1.3)	33 (1.2)	
Urinary tract infection	212 (7.7)	2139 (8.4)		205 (7.7)	199 (7.5)	
Venous thromboembolism	68 (2.5)	585 (2.3)		65 (2.5)	57 (2.2)	
**Hospital blood glucose values, mean (SD)**						
Highest glucose	310.6 (110.6)	235.7 (85.3)	0.76	304 (105.5)	306.4 (109.4)	0.02
Lowest glucose	107.7 (45.2)	102.4 (32.3)	0.13	108 (44.2)	107.7 (43.4)	0.01
Standard deviation of hospital glucose recordings	57.4 (29.5)	39.6 (22.8)	0.68	55.8 (28.3)	56.4 (30.0)	0.02
**Laboratory values at discharge, mean (SD)** [c]						
Glucose, mg/dL	181.8 (71.4)	156.5 (57.7)	0.39	180.7 (70.3)	181.0 (68.9)	0.00
Hemoglobin, g/dL	12.0 (2.0)	12.1 (2.0)	0.04	12.0 (2.0)	12.0 (2.0)	0.00
Platelets, k/mm3	223.2 (86.4)	212.3 (82.5)	0.13	222.2 (85.8)	223.9 (94.3)	0.02

© 2021 Anderson TS et al. *JAMA Network Open.*

Sodium, mEQ/L	137.6 (3.2)	138.0 (3.2)	0.12	137.7 (3.2)	137.6 (3.2)	0.01
Potassium, mEQ/L	4.1 (0.4)	4.1 (0.4)	0.03	4.1 (0.4)	4.1 (0.5)	0.01
Blood urea nitrogen, mg/dL	25.1 (14.4)	23.0 (13.1)	0.15	25.1 (14.4)	25.1 (14.8)	0.00
Carbon dioxide, mEQ/L	27.2 (3.9)	27.0 (3.9)	0.05	27.2 (3.9)	27.1 (4.0)	0.02
Estimated glomerular filtration rate, mL/min per 1.73 m2	66.9 (26.3)	69.6 (26.9)	0.10	67 (26.2)	66.9 (26.9)	0.00
**Received inpatient steroids, No. (%)**	509 (18.4)	3459 (13.6)	0.13	491 (18.5)	444 (16.8)	0.05
**Other medication classes used at hospital admission, No. (%)**[b]						
Antihistamines	351 (12.7)	3869 (15.2)	0.07	341 (12.9)	315 (11.9)	0.03
Antimicrobials	92 (3.3)	970 (3.8)	0.03	90 (3.4)	95 (3.6)	0.01
Antineoplastics	53 (1.9)	583 (2.3)	0.03	52 (2.0)	54 (2.0)	0.01
Autonomic medications	48 (1.7)	503 (2.0)	0.02	45 (1.7)	42 (1.6)	0.01
Anticoagulants	313 (11.3)	3957 (15.6)	0.12	298 (11.3)	298 (11.3)	0.00
Platelet aggregation inhibitors	284 (10.3)	2759 (10.8)	0.02	278 (10.5)	306 (11.6)	0.03
Other central nervous system medications	192 (6.9)	2080 (8.2)	0.05	185 (7.0)	164 (6.2)	0.03
Non-opioid analgesics	781 (28.2)	7711 (30.3)	0.05	749 (28.3)	765 (28.9)	0.01
Opioid analgesics	512 (18.5)	5455 (21.5)	0.07	498 (18.8)	502 (19.0)	0.00
Sedative/hypnotics	261 (9.4)	2905 (11.4)	0.07	256 (9.7)	256 (9.7)	0.00
Anticonvulsants	507 (18.3)	4960 (19.5)	0.03	484 (18.3)	459 (17.3)	0.02
Antdepressants	676 (24.4)	6839 (26.9)	0.06	653 (24.7)	636 (24.0)	0.01
Antipsychotics	102 (3.7)	1185 (4.7)	0.05	101 (3.8)	98 (3.7)	0.01
Digitalis glycosides	114 (4.1)	1242 (4.9)	0.04	106 (4.0)	106 (4.0)	0.00
Beta blockers	1379 (49.8)	13729 (54)	0.08	1328 (50.2)	1336 (50.5)	0.01
Alpha blockers	736 (26.6)	7969 (31.3)	0.10	721 (27.2)	745 (28.1)	0.02
Calcium channel blockers	836 (30.2)	7908 (31.1)	0.02	807 (30.5)	817 (30.9)	0.01
Antianginals	448 (16.2)	4215 (16.6)	0.01	433 (16.4)	424 (16.0)	0.01
Antiarrhythmics	51 (1.8)	577 (2.3)	0.03	49 (1.9)	45 (1.7)	0.01
Antilipemics	1791 (64.7)	17685 (69.5)	0.10	1726 (65.2)	1749 (66.0)	0.02
Other antihypertensives	283 (10.2)	2561 (10.1)	0.01	273 (10.3)	285 (10.8)	0.01
Diuretics	546 (19.7)	5472 (21.5)	0.04	531 (20.1)	503 (19.0)	0.03
Loop diuretics	734 (26.5)	6779 (26.7)	0.00	705 (26.6)	714 (27.0)	0.01
Angiotension converting enzyme inhibitors	1199 (43.3)	11465 (45.1)	0.04	1157 (43.7)	1161 (43.8)	0.00
Angiotensin II receptor blockers	354 (12.8)	3550 (14.0)	0.03	342 (12.9)	349 (13.2)	0.01
Gastrointenstinal medications	1247 (45.1)	13048 (51.3)	0.13	1205 (45.5)	1200 (45.3)	0.00
Genitourinary medications	291 (10.5)	3148 (12.4)	0.06	286 (10.8)	294 (11.1)	0.01
Hormones	629 (22.7)	6913 (27.2)	0.10	607 (22.9)	592 (22.4)	0.01
Musculoskeletal medications	595 (21.5)	6059 (23.8)	0.06	579 (21.9)	562 (21.2)	0.02
Antiasthma/brochodilators	47 (1.7)	517 (2.0)	0.02	46 (1.7)	43 (1.6)	0.01
Systemic decongestants and antitussives	124 (4.5)	1272 (5.0)	0.02	120 (4.5)	119 (4.5)	0.00
**Comorbidities, No. (%)** [d]						
Heart failure	1059 (38.3)	9263 (36.4)	0.04	1008 (38.1)	1019 (38.5)	0.01
Acute myocardial infarction	273 (9.9)	2387 (9.4)	0.02	260 (9.8)	280 (10.6)	0.02

© 2021 Anderson TS et al. *JAMA Network Open.*

Unstable angina and other acute ischemic heart disease	174 (6.3)	1783 (7.0)	0.03	164 (6.2)	163 (6.2)	0.00
Chronic angina and coronary artery disease	1457 (52.6)	13202 (51.9)	0.01	1393 (52.6)	1434 (54.2)	0.03
Valvular/rheumatic heart disease	381 (13.8)	4257 (16.7)	0.08	373 (14.1)	383 (14.5)	0.01
Other cardiac disease including congenital heart and hypertensive disease	1693 (61.2)	15627 (61.5)	0.01	1620 (61.2)	1640 (61.9)	0.02
Arrhythmias and conduction disorders	1272 (46.0)	12821 (50.4)	0.09	1221 (46.1)	1230 (46.5)	0.01
Pleural effusion/pneumothorax	210 (7.6)	2067 (8.1)	0.02	199 (7.5)	194 (7.3)	0.01
Chest pain	684 (24.7)	6736 (26.5)	0.04	662 (25.0)	650 (24.5)	0.01
Syncope	161 (5.8)	1777 (7.0)	0.05	157 (5.9)	161 (6.1)	0.01
Acute stroke/transient ischemic attack	483 (17.4)	4173 (16.4)	0.03	454 (17.1)	494 (18.7)	0.04
Pulmonary embolism/deep venous thrombosis	166 (6.0)	1567 (6.2)	0.01	157 (5.9)	146 (5.5)	0.02
Other peripheral vascular disease	610 (22.0)	5222 (20.5)	0.04	571 (21.6)	586 (22.1)	0.01
Cardio-respiratory failure	66 (2.4)	591 (2.3)	0.00	64 (2.4)	69 (2.6)	0.01
Chronic obstructive pulmonary disease/asthma	1165 (42.1)	10892 (42.8)	0.02	1117 (42.2)	1108 (41.8)	0.01
Pneumonia including aspiration pneumonitis	638 (23.0)	5547 (21.8)	0.03	612 (23.1)	592 (22.4)	0.02
Septicemia/shock	200 (7.2)	1902 (7.5)	0.01	192 (7.3)	181 (6.8)	0.02
Urinary tract infection and urinary system complaints	529 (19.1)	5065 (19.9)	0.02	502 (19.0)	475 (17.9)	0.03
Cellulitis	540 (19.5)	4833 (19.0)	0.01	506 (19.1)	504 (19.0)	0.00
Clostridium difficile-associated infection	27 (1.0)	240 (0.9)	0.00	25 (0.9)	27 (1.0)	0.01
Renal disorders including renal failure and fluid, electrolyte, and acid-base abnormalities	1457 (52.6)	12681 (49.9)	0.06	1376 (52.0)	1344 (50.8)	0.02
Anemia	882 (31.9)	8352 (32.8)	0.02	846 (31.9)	839 (31.7)	0.01
Gastrointestinal hemorrhage	199 (7.2)	1986 (7.8)	0.02	193 (7.3)	189 (7.1)	0.01
Fibrosis of lung and other chronic lung disorders	87 (3.1)	818 (3.2)	0.00	81 (3.1)	88 (3.3)	0.02
Hip fracture	22 (0.8)	201 (0.8)	0.00	22 (0.8)	19 (0.7)	0.01
Complications of care	374 (13.5)	3703 (14.6)	0.03	360 (13.6)	353 (13.3)	0.01
Other lung disorders including acute, congenital, and unspecified lung abnormalities	1108 (40.0)	10796 (42.5)	0.05	1065 (40.2)	1068 (40.3)	0.00
Primary cancer of the trachea, bronchus, lung, and pleura	72 (2.6)	757 (3.0)	0.02	69 (2.6)	67 (2.5)	0.00
Other infections	1414 (51.1)	13427 (52.8)	0.03	1349 (50.9)	1337 (50.5)	0.01
Cancer	544 (19.7)	5972 (23.5)	0.09	528 (19.9)	505 (19.1)	0.02
Endocrine/metabolic	2603 (94.0)	24006 (94.4)	0.02	2495 (94.2)	2483 (93.8)	0.02
Liver disease	230 (8.3)	2217 (8.7)	0.01	218 (8.2)	226 (8.5)	0.01
Digestive (Gastrointestinal)	1547 (55.9)	15465 (60.8)	0.10	1484 (56.0)	1504 (56.8)	0.02

© 2021 Anderson TS et al. *JAMA Network Open.*

Musculoskeletal	2115 (76.4)	20332 (80.0)	0.09	2016 (76.1)	2045 (77.2)	0.03
Other hematologic	411 (14.8)	3783 (14.9)	0.00	391 (14.8)	401 (15.1)	0.01
Cognitive	299 (10.8)	2901 (11.4)	0.02	282 (10.6)	311 (11.7)	0.03
Substance abuse	301 (10.9)	2526 (9.9)	0.03	280 (10.6)	281 (10.6)	0.00
Psychiatric	1214 (43.9)	11270 (44.3)	0.01	1153 (43.5)	1166 (44)	0.01
Development disorders	31 (1.1)	256 (1.0)	0.01	29 (1.1)	31 (1.2)	0.01
Spinal cord	81 (2.9)	757 (3.0)	0.00	78 (2.9)	87 (3.3)	0.02
Neuromuscular	1377 (49.7)	12834 (50.5)	0.01	1311 (49.5)	1328 (50.2)	0.01
Non-acute cerebrovascular disease	410 (14.8)	3842 (15.1)	0.01	393 (14.8)	411 (15.5)	0.02
Other pulmonary	61 (2.2)	639 (2.5)	0.02	59 (2.2)	53 (2.0)	0.02
Ophthalmologic	2107 (76.1)	20232 (79.6)	0.08	2016 (76.1)	2048 (77.3)	0.03
Ear, nose, and throat	1187 (42.9)	12909 (50.8)	0.16	1157 (43.7)	1152 (43.5)	0.00
Other renal	502 (18.1)	4553 (17.9)	0.01	476 (18.0)	481 (18.2)	0.00
Genitourinary	1418 (51.2)	13677 (53.8)	0.05	1356 (51.2)	1353 (51.1)	0.00
Other dermatologic	1296 (46.8)	12558 (49.4)	0.05	1237 (46.7)	1245 (47.0)	0.01
Injury excluding hip fracture	608 (22.0)	6070 (23.9)	0.05	579 (21.9)	577 (21.8)	0.00
Symptoms (major and minor) excluding respiratory	1031 (37.2)	10272 (40.4)	0.06	989 (37.3)	962 (36.3)	0.02
Miscellaneous surgical and non-surgical procedures	2720 (98.3)	25035 (98.4)	0.01	2601 (98.2)	2597 (98.1)	0.01
Durable medical equipment	779 (28.1)	7569 (29.8)	0.04	747 (28.2)	754 (28.5)	0.01
Other	2209 (79.8)	20643 (81.2)	0.03	2112 (79.8)	2121 (80.1)	0.01
Skin ulcer including decubitus ulcer	346 (12.5)	2582 (10.2)	0.07	321 (12.1)	322 (12.2)	0.00

Abbreviations: SD, standard deviation; BP, blood pressure; ED, emergency department

[a] Fewer than 8% of any covariate was missing. Missing data were imputed using the fully conditional specification method and 20 imputation sets. Missing data included: BMI (n= 2,282), outpatient blood pressure (n= 1,594), pre-admission estimated glomerular filtration rate (n=1,560), inpatient glucose (n=2,012), discharge hemoglobin (n=1,705), discharge potassium (n=961), discharge sodium (n=883), discharge blood urea nitrogen (n=1,337), discharge carbon dioxide (n=1,201), discharge platelets (n=1,235), discharge estimated glomerular filtration rate (n=1,476).

[b] All medications classified using VA drug class coding. Combination medications were split into component parts. Topical, inhaled, otic, and optic medications were excluded.

[c] Laboratory data collected from day of index hospitalization discharge or during index hospitalization up to 2 days prior to day of discharge

[d] Comorbidities include both secondary discharge diagnoses from index hospitalization and preadmission diagnoses from the year preceding index hospitalization.

© 2021 Anderson TS et al. *JAMA Network Open.*

eTable 4. Difference in Differences Analysis of Hemoglobin A_{1c} Values One Year Following Discharged With or Without Diabetes Medication Intensifications

**HbA_{1c}, Mean (95% CI), % [a]**	**Received Discharge Intensifications (N=2,157)**	**No Discharge Intensifications (N=2,057)**
Baseline	7.91 (7.84 to 7.98)	7.91 (7.84 to 7.97)
Post-baseline [b]	7.72 (7.65 to 7.79)	7.70 (7.68 to 7.77)
Pre-post change	-0.19 (-0.29 to -0.10)	-0.21 (-0.31 to -0.11)
Difference-in-differences estimate [c]	0.02 (-0.12 to 0.16)	

Abbreviations: SD, standard deviation, HbA_{1c}, hemoglobin A1c

[a] The results are from the 4,215 patients in the propensity-matched cohort who met the inclusion criteria for the HbA_{1c} analysis (outpatient HbA_{1c} measured between 6 months and 18 months following index hospitalization discharge). Mean (SD) number of days between baseline and postbaseline measure 278 (80) days for intensified group and 282 (82) days for not intensified group.

[b] The post-discharge HbA_{1c} was assessed prior to censoring as the HbA_{1c} recorded at the closest date to 1 year following index hospitalization discharge, within the range for 6 to 18 months following discharge.

[c] Calculated as the pre-post change in HbA_{1c} for the intensified regimen group minus the pre-post change in HbA_{1c} for the not intensified group.

eTable 5. Baseline Characteristics Before and After Propensity Score Matching, Controlled Baseline HbA_{1c} Subgroup

	**Before Propensity Score Matching**			**After Propensity Score Matching**		
**Characteristic [a]**	**Intensified (N= 1,341)**	**Not Intensified (N= 18,926)**	**SMD**	**Intensified (N= 1,336)**	**Not Intensified (N= 1,336)**	**SMD**
Age, mean (SD), y	73.4 (7.5)	74.0 (7.7)	0.07	73.4 (7.5)	73.3 (7.3)	0.01
Male sex, No. (%)	1322 (98.6)	18576 (98.2)	0.03	1317 (98.6)	1312 (98.2)	0.03
Race/ethnicity, No. (%)			0.05			0.02
White	1062 (79.2)	15251 (80.6)		1059 (79.3)	1065 (79.7)	
Black	207 (15.4)	2760 (14.6)		206 (15.4)	203 (15.2)	
Hispanic	15 (1.1)	260 (1.4)		15 (1.1)	16 (1.2)	
Other	57 (4.3)	655 (3.5)		56 (4.2)	52 (3.9)	
Median zip code household income, mean (SD), $	27063.5 (45097.4)	26950.7 (43394.2)	0.00	27116.9 (45166.1)	26440 (41195.5)	0.02
VISN, No. (%)			0.23			0.09
New England Health Care System	49 (3.7)	881 (4.7)		49 (3.7)	44 (3.3)	
Healthcare New York/New Jersey	82 (6.1)	964 (5.1)		81 (6.1)	69 (5.2)	
VISN4 Network	57 (4.3)	830 (4.4)		57 (4.3)	55 (4.1)	
Capitol Healthcare Network	49 (3.7)	485 (2.6)		49 (3.7)	55 (4.1)	
Mid-Atlantic Health Care Network	78 (5.8)	1187 (6.3)		78 (5.8)	73 (5.5)	
Southeast Network	84 (6.3)	868 (4.6)		84 (6.3)	78 (5.8)	
Sunshine Healthcare Network	108 (8.1)	1887 (10)		108 (8.1)	106 (7.9)	
MidSouth Healthcare Network	80 (6)	1356 (7.2)		80 (6)	78 (5.8)	
VISN 10 Healthcare System	54 (4)	618 (3.3)		54 (4)	68 (5.1)	
Great Lakes Healthcare System	143 (10.7)	1765 (9.3)		140 (10.5)	140 (10.5)	
Heartland Network	59 (4.4)	971 (5.1)		59 (4.4)	65 (4.9)	
South Central VA Healthcare Network	160 (11.9)	1904 (10.1)		160 (12)	158 (11.8)	
Heart of Texas Healthcare System	35 (2.6)	972 (5.1)		35 (2.6)	36 (2.7)	
Southwest Healthcare Network	41 (3.1)	739 (3.9)		41 (3.1)	36 (2.7)	
Rocky Mountain Network	36 (2.7)	445 (2.4)		36 (2.7)	36 (2.7)	
Northwest Health Network	42 (3.1)	653 (3.5)		42 (3.1)	46 (3.4)	
Sierra Pacific Network	42 (3.1)	724 (3.8)		42 (3.1)	42 (3.1)	
Desert Pacific Healthcare Network	86 (6.4)	995 (5.3)		86 (6.4)	87 (6.5)	
Midwest Healthcare Network	56 (4.2)	682 (3.6)		55 (4.1)	64 (4.8)	
**Pre-admission clinical characteristics, mean (SD)**						
Body mass index, kg/m^2	31.0 (6.7)	30.8 (6.5)	0.03	31.0 (6.7)	31.3 (7)	0.04
Systolic BP, mmHg	133.8 (17.9)	132.6 (17.4)	0.07	133.8 (17.9)	134.2 (18.2)	0.02

© 2021 Anderson TS et al. *JAMA Network Open.*

Diastolic BP, mmHg	72.3 (10.4)	72.1 (10.2)	0.02	72.3 (10.4)	72.5 (10.4)	0.01
Frailty score	0.2 (0.1)	0.2 (0.1)	0.04	0.2 (0.1)	0.2 (0.1)	0.03
**Pre-admission diabetes management**						
Hemoglobin A1c, mean (SD), d	6.8 (0.5)	6.6 (0.5)	0.45	6.8 (0.5)	6.8 (0.5)	0.01
Estimated glomerular filtration rate, mean (SD), mL/min per 1.73 m2	64.9 (23.9)	67 (24.4)	0.09	64.9 (23.9)	64.3 (24)	0.03
Any hypoglycemia hospitalizations in prior year, No. (%)	21 (1.6)	142 (0.8)	0.08	20 (1.5)	18 (1.3)	0.01
Number of admission diabetes medications, No. (%)			0.30			0.05
0	495 (36.9)	4593 (24.3)		492 (36.8)	519 (38.8)	
1	573 (42.7)	10502 (55.5)		572 (42.8)	564 (42.2)	
2	241 (18)	3483 (18.4)		240 (18)	220 (16.5)	
>3	32 (2.4)	348 (1.8)		32 (2.4)	33 (2.4)	
Admission diabetes medication classes, No. (%) [b]						
Metformin	511 (38.1)	9321 (49.2)	0.23	511 (38.2)	488 (36.5)	0.04
Sulfonylureas	538 (40.1)	8229 (43.5)	0.07	536 (40.1)	518 (38.8)	0.03
Thiazolidinediones	58 (4.3)	463 (2.4)	0.10	57 (4.3)	57 (4.3)	0.00
α-glucosidase inhibitors	21 (1.6)	233 (1.2)	0.03	21 (1.6)	20 (1.5)	0.01
Dipeptidyl peptidase-4 inhibitors	17 (1.3)	185 (1)	0.03	17 (1.3)	13 (1)	0.03
Other classes	2 (0.1)	28 (0.1)	0.00	2 (0.1)	0 (0)	0.05
Preadmission diabetes medication adherence			0.28			0.05
0 medications	495 (36.9)	4593 (24.3)		492 (36.8)	519 (38.8)	
PDC ≤ 80%	265 (19.8)	3998 (21.1)		264 (19.8)	247 (18.5)	
PDC > 80%	581 (43.3)	10335 (54.6)		580 (43.4)	570 (42.7)	
Diabetes medication classes used in prior year, No. (%)						
Metformin	681 (50.8)	10186 (53.8)	0.06	678 (50.7)	663 (49.6)	0.02
Sulfonylureas	711 (53)	8888 (47)	0.12	707 (52.9)	698 (52.2)	0.01
Thiazolidinediones	75 (5.6)	564 (3)	0.13	74 (5.5)	80 (6)	0.02
α-glucosidase inhibitors	30 (2.2)	284 (1.5)	0.05	30 (2.2)	31 (2.3)	0.01
Dipeptidyl peptidase-4 inhibitors	15 (1.1)	133 (0.7)	0.04	15 (1.1)	11 (0.8)	0.03
Insulins	217 (16.2)	716 (3.8)	0.42	214 (16)	210 (15.7)	0.01
Other classes	4 (0.3)	44 (0.2)	0.01	4 (0.3)	2 (0.1)	0.03
**Pre-admission healthcare use, No. (%)**						
Any Medicare utilization in prior year, No. (%)	419 (31.2)	5741 (30.3)	0.02	418 (31.3)	429 (32.1)	0.02
No. ED visits in the year preceding index hospitalization			0.10			0.10
0	674 (50.3)	9421 (49.8)		671 (50.2)	680 (50.9)	
1	346 (25.8)	4527 (23.9)		345 (25.8)	335 (25.1)	
2	175 (13)	2346 (12.4)		175 (13.1)	173 (12.9)	

© 2021 Anderson TS et al. *JAMA Network Open.*

≥3	146 (10.9)	2632 (13.9)		145 (10.9)	148 (11.1)	
No. hospitalizations in the year preceding index hospitalization			0.05			0.06
0	941 (70.2)	13362 (70.6)		937 (70.1)	938 (70.2)	
1	229 (17.1)	3392 (17.9)		229 (17.1)	239 (17.9)	
2	90 (6.7)	1252 (6.6)		90 (6.7)	73 (5.5)	
≥3	81 (6)	920 (4.9)		80 (6)	86 (6.4)	
Admission medication count, mean (SD)	8.6 (4.7)	9.3 (4.8)	0.15	8.6 (4.7)	8.3 (4.9)	0.05
Polypharmacy (>8 medications), No. (%)	765 (57)	12156 (64.2)	0.15	765 (57.3)	746 (55.8)	0.03
**Index hospitalization characteristics**						
Length of stay, mean (SD), d	6.6 (6.4)	5.2 (5.5)	0.24	6.6 (6.4)	6.8 (8.2)	0.02
Year of hospitalization, No. (%)			0.16			0.14
2011	360 (26.8)	4365 (23.1)		358 (26.8)	381 (28.5)	
2012	266 (19.8)	3400 (18)		266 (19.9)	324 (24.3)	
2013	236 (17.6)	3182 (16.8)		235 (17.6)	189 (14.1)	
2014	209 (15.6)	2997 (15.8)		207 (15.5)	191 (14.3)	
2015	150 (11.2)	2888 (15.3)		150 (11.2)	143 (10.7)	
2016	120 (8.9)	2094 (11.1)		120 (9)	108 (8.1)	
Training hospital, No. (%)	1184 (88.3)	16926 (89.4)	0.04	1181 (88.4)	1201 (89.9)	0.05
**Discharge diagnoses, No. (%)**			0.22			0.09
Arrhythmia	6 (0.4)	279 (1.5)		6 (0.4)	8 (0.6)	
Asthma	12 (0.9)	100 (0.5)		12 (0.9)	10 (0.7)	
Chronic obstructive pulmonary disease	163 (12.2)	1693 (8.9)		163 (12.2)	168 (12.6)	
Chest pain	40 (3)	722 (3.8)		40 (3)	33 (2.5)	
Conduction disorders	108 (8.1)	2091 (11)		108 (8.1)	103 (7.7)	
Coronary artery disease	173 (12.9)	2064 (10.9)		172 (12.9)	172 (12.9)	
Acute coronary syndrome	71 (5.3)	1044 (5.5)		70 (5.2)	74 (5.5)	
Heart failure	236 (17.6)	2882 (15.2)		234 (17.5)	236 (17.7)	
Heart valve disorders	41 (3.1)	470 (2.5)		41 (3.1)	52 (3.9)	
Pneumonia	144 (10.7)	2148 (11.3)		144 (10.8)	140 (10.5)	
Sepsis	32 (2.4)	591 (3.1)		32 (2.4)	40 (3)	
Skin infection	102 (7.6)	1643 (8.7)		102 (7.6)	106 (7.9)	
Stroke	65 (4.8)	883 (4.7)		65 (4.9)	61 (4.6)	
Transient ischemic attack	18 (1.3)	312 (1.6)		18 (1.3)	19 (1.4)	
Urinary tract infection	101 (7.5)	1580 (8.3)		100 (7.5)	88 (6.6)	
Venous thromboembolism	29 (2.2)	424 (2.2)		29 (2.2)	26 (1.9)	
**Hospital blood glucose values, mean (SD)**						
Highest glucose	284.5 (104.5)	222.1 (79)	0.67	283.5 (103.3)	281 (102.9)	0.02
Lowest glucose	102.7 (39.1)	99.6 (28.5)	0.09	102.8 (39.1)	99.8 (34.6)	0.08
Standard deviation of hospital glucose recordings	51.3 (27.1)	36.3 (20.9)	0.62	50.9 (26.6)	51.1 (28)	0.01
**Laboratory values at discharge, mean (SD) [c]**						
Glucose, mg/dL	170.1 (66.3)	148.2 (52)	0.37	169.7 (65.5)	166.2 (63.5)	0.06
Hemoglobin, g/dL	11.9 (2)	12.1 (2)	0.08	11.9 (2)	11.9 (1.9)	0.03

© 2021 Anderson TS et al. *JAMA Network Open.*

Platelets, k/mm3	221.1 (86.4)	210.8 (82.2)	0.12	221 (86.4)	221.1 (87.7)	0.00
Sodium, mEQ/L	137.7 (3.3)	138.1 (3.2)	0.12	137.7 (3.3)	137.6 (3.4)	0.04
Potassium, mEQ/L	4.1 (0.4)	4.1 (0.4)	0.04	4.1 (0.4)	4.2 (0.4)	0.04
Blood urea nitrogen, mg/dL	26.1 (14.2)	23 (12.9)	0.22	26 (14.1)	26.5 (16.2)	0.03
Carbon dioxide, mEQ/L	27.4 (3.9)	27 (3.9)	0.09	27.4 (3.9)	27.3 (4.1)	0.03
Estimated glomerular filtration rate, mL/min per 1.73 m2	66.2 (26.1)	69.7 (26.6)	0.13	66.3 (26.1)	66.2 (26.5)	0.00
**Other medication classes used at hospital admission, No. (%)**[b]						
Antihistamines	198 (14.8)	3064 (16.2)	0.04	198 (14.8)	187 (14)	0.02
Antimicrobials	44 (3.3)	771 (4.1)	0.04	44 (3.3)	44 (3.3)	0.00
Antineoplastics	27 (2)	449 (2.4)	0.02	27 (2)	22 (1.6)	0.03
Autonomic medications	35 (2.6)	399 (2.1)	0.03	35 (2.6)	33 (2.5)	0.01
Anticoagulants	169 (12.6)	3092 (16.3)	0.11	169 (12.6)	160 (12)	0.02
Platelet aggregation inhibitors	155 (11.6)	2106 (11.1)	0.01	155 (11.6)	169 (12.6)	0.03
Other central nervous system medications	93 (6.9)	1604 (8.5)	0.06	93 (7)	99 (7.4)	0.02
Non-opioid analgesics	394 (29.4)	5920 (31.3)	0.04	393 (29.4)	378 (28.3)	0.02
Opioid analgesics	267 (19.9)	4299 (22.7)	0.07	267 (20)	246 (18.4)	0.04
Sedative/hypnotics	149 (11.1)	2292 (12.1)	0.03	149 (11.2)	131 (9.8)	0.04
Anticonvulsants	249 (18.6)	3908 (20.6)	0.05	249 (18.6)	228 (17.1)	0.04
Antdepressants	349 (26)	5362 (28.3)	0.05	348 (26)	331 (24.8)	0.03
Antipsychotics	63 (4.7)	943 (5)	0.01	63 (4.7)	53 (4)	0.04
Digitalis glycosides	64 (4.8)	931 (4.9)	0.01	64 (4.8)	84 (6.3)	0.07
Beta blockers	712 (53.1)	10576 (55.9)	0.06	710 (53.1)	710 (53.1)	0.00
Alpha blockers	396 (29.5)	6264 (33.1)	0.08	396 (29.6)	360 (26.9)	0.06
Calcium channel blockers	443 (33)	6154 (32.5)	0.01	441 (33)	470 (35.2)	0.05
Antianginals	228 (17)	3238 (17.1)	0.00	228 (17.1)	233 (17.4)	0.01
Antiarrhythmics	29 (2.2)	473 (2.5)	0.02	29 (2.2)	30 (2.2)	0.01
Antilipemics	916 (68.3)	13609 (71.9)	0.08	913 (68.3)	899 (67.3)	0.02
Other antihypertensives	154 (11.5)	1986 (10.5)	0.03	153 (11.5)	149 (11.2)	0.01
Diuretics	274 (20.4)	4218 (22.3)	0.05	274 (20.5)	286 (21.4)	0.02
Loop diuretics	410 (30.6)	5268 (27.8)	0.06	409 (30.6)	402 (30.1)	0.01
Angiotension converting enzyme inhibitors	620 (46.2)	8867 (46.9)	0.01	619 (46.3)	620 (46.4)	0.00
Angiotensin II receptor blockers	178 (13.3)	2722 (14.4)	0.03	176 (13.2)	173 (12.9)	0.01
Gastrointenstinal medications	678 (50.6)	10225 (54)	0.07	677 (50.7)	663 (49.6)	0.02
Genitourinary medications	157 (11.7)	2419 (12.8)	0.03	156 (11.7)	146 (10.9)	0.02
Hormones	340 (25.4)	5416 (28.6)	0.07	339 (25.4)	304 (22.8)	0.06
Musculoskeletal medications	329 (24.5)	4848 (25.6)	0.02	328 (24.6)	345 (25.8)	0.03
Antiasthma/brochodilators	31 (2.3)	419 (2.2)	0.01	31 (2.3)	28 (2.1)	0.02
Systemic decongestants and antitussives	74 (5.5)	1002 (5.3)	0.01	74 (5.5)	72 (5.4)	0.01
**Comorbidities, No. (%)** [d]						
Heart failure	530 (39.5)	6943 (36.7)	0.06	527 (39.4)	538 (40.3)	0.02
Acute myocardial infarction	129 (9.6)	1697 (9)	0.02	128 (9.6)	140 (10.5)	0.03
Unstable angina and other acute ischemic heart disease	87 (6.5)	1318 (7)	0.02	86 (6.4)	82 (6.1)	0.01

© 2021 Anderson TS et al. *JAMA Network Open.*

Chronic angina and coronary artery disease	757 (56.5)	9826 (51.9)	0.09	754 (56.4)	791 (59.2)	0.06
Valvular/rheumatic heart disease	205 (15.3)	3304 (17.5)	0.06	205 (15.3)	209 (15.6)	0.01
Other cardiac disease including congenital heart and hypertensive disease	847 (63.2)	11652 (61.6)	0.03	843 (63.1)	846 (63.3)	0.00
Arrhythmias and conduction disorders	666 (49.7)	9708 (51.3)	0.03	664 (49.7)	663 (49.6)	0.00
Pleural effusion/pneumothorax	121 (9)	1547 (8.2)	0.03	121 (9.1)	142 (10.6)	0.05
Chest pain	362 (27)	5118 (27)	0.00	360 (26.9)	351 (26.3)	0.02
Syncope	82 (6.1)	1386 (7.3)	0.05	82 (6.1)	75 (5.6)	0.02
Acute stroke/transient ischemic attack	230 (17.2)	3053 (16.1)	0.03	227 (17)	222 (16.6)	0.01
Pulmonary embolism/deep venous thrombosis	77 (5.7)	1145 (6)	0.01	77 (5.8)	74 (5.5)	0.01
Other peripheral vascular disease	294 (21.9)	3868 (20.4)	0.04	290 (21.7)	311 (23.3)	0.04
Cardio-respiratory failure	33 (2.5)	443 (2.3)	0.01	32 (2.4)	36 (2.7)	0.02
Chronic obstructive pulmonary disease/asthma	640 (47.7)	8341 (44.1)	0.07	638 (47.8)	658 (49.3)	0.03
Pneumonia including aspiration pneumonitis	353 (26.3)	4236 (22.4)	0.09	351 (26.3)	345 (25.8)	0.01
Septicemia/shock	103 (7.7)	1422 (7.5)	0.01	102 (7.6)	94 (7)	0.02
Urinary tract infection and urinary system complaints	263 (19.6)	3816 (20.2)	0.01	262 (19.6)	241 (18)	0.04
Cellulitis	247 (18.4)	3555 (18.8)	0.01	246 (18.4)	248 (18.6)	0.00
Clostridium difficile-associated infection	14 (1)	182 (1)	0.01	14 (1)	14 (1)	0.00
Renal disorders including renal failure and fluid, electrolyte, and acid-base abnormalities	735 (54.8)	9514 (50.3)	0.09	730 (54.6)	735 (55)	0.01
Anemia	457 (34.1)	6422 (33.9)	0.00	455 (34.1)	439 (32.9)	0.03
Gastrointestinal hemorrhage	112 (8.4)	1544 (8.2)	0.01	112 (8.4)	120 (9)	0.02
Fibrosis of lung and other chronic lung disorders	57 (4.3)	618 (3.3)	0.05	57 (4.3)	54 (4)	0.01
Hip fracture	12 (0.9)	163 (0.9)	0.00	12 (0.9)	14 (1)	0.02
Complications of care	206 (15.4)	2780 (14.7)	0.02	205 (15.3)	205 (15.3)	0.00
Other lung disorders including acute, congenital, and unspecified lung abnormalities	595 (44.4)	8164 (43.1)	0.02	593 (44.4)	579 (43.3)	0.02
Primary cancer of the trachea, bronchus, lung, and pleura	43 (3.2)	589 (3.1)	0.01	43 (3.2)	34 (2.5)	0.04
Other infections	716 (53.4)	10084 (53.3)	0.00	713 (53.4)	713 (53.4)	0.00
Cancer	298 (22.2)	4611 (24.4)	0.05	295 (22.1)	246 (18.4)	0.09
Endocrine/metabolic	1277 (95.2)	17908 (94.6)	0.03	1272 (95.2)	1264 (94.6)	0.03
Liver disease	111 (8.3)	1675 (8.9)	0.02	110 (8.2)	125 (9.4)	0.04
Digestive (Gastrointestinal)	810 (60.4)	11775 (62.2)	0.04	807 (60.4)	827 (61.9)	0.03
Musculoskeletal	1079 (80.5)	15349 (81.1)	0.02	1075 (80.5)	1062 (79.5)	0.02
Other hematologic	219 (16.3)	2930 (15.5)	0.02	216 (16.2)	203 (15.2)	0.03

© 2021 Anderson TS et al. *JAMA Network Open.*

Cognitive	148 (11)	2082 (11)	0.00	147 (11)	169 (12.6)	0.05
Substance abuse	140 (10.4)	1972 (10.4)	0.00	140 (10.5)	133 (10)	0.02
Psychiatric	579 (43.2)	8491 (44.9)	0.03	577 (43.2)	564 (42.2)	0.02
Development disorders	15 (1.1)	189 (1)	0.01	15 (1.1)	8 (0.6)	0.06
Spinal cord	43 (3.2)	562 (3)	0.01	43 (3.2)	41 (3.1)	0.01
Neuromuscular	649 (48.4)	9646 (51)	0.05	647 (48.4)	648 (48.5)	0.00
Non-acute cerebrovascular disease	225 (16.8)	2902 (15.3)	0.04	222 (16.6)	219 (16.4)	0.01
Other pulmonary	33 (2.5)	491 (2.6)	0.01	33 (2.5)	28 (2.1)	0.03
Ophthalmologic	1033 (77)	15121 (79.9)	0.07	1029 (77)	1020 (76.3)	0.02
Ear, nose, and throat	627 (46.8)	9780 (51.7)	0.10	626 (46.9)	606 (45.4)	0.03
Other renal	257 (19.2)	3400 (18)	0.03	256 (19.2)	250 (18.7)	0.01
Genitourinary	716 (53.4)	10409 (55)	0.03	712 (53.3)	691 (51.7)	0.03
Other dermatologic	658 (49.1)	9561 (50.5)	0.03	656 (49.1)	636 (47.6)	0.03
Injury excluding hip fracture	306 (22.8)	4538 (24)	0.03	305 (22.8)	291 (21.8)	0.03
Symptoms (major and minor) excluding respiratory	517 (38.6)	7790 (41.2)	0.05	516 (38.6)	516 (38.6)	0.00
Miscellaneous surgical and non-surgical procedures	1329 (99.1)	18663 (98.6)	0.05	1324 (99.1)	1319 (98.7)	0.04
Durable medical equipment	410 (30.6)	5758 (30.4)	0.00	408 (30.5)	398 (29.8)	0.02
Other	1114 (83.1)	15470 (81.7)	0.04	1110 (83.1)	1070 (80.1)	0.08
Skin ulcer including decubitus ulcer	165 (12.3)	1829 (9.7)	0.08	163 (12.2)	150 (11.2)	0.03

© 2021 Anderson TS et al. *JAMA Network Open.*

eTable 6. Baseline Characteristics Before and After Propensity Score Matching, Elevated Baseline HbA_{1c} Subgroup

	**Before Propensity Score Matching**			**After Propensity Score Matching**		
**Characteristic** [a]	**Intensified (N= 1,350)**	**Not Intensified (N= 5,711)**	**SMD**	**Intensified (N= 1,262)**	**Not Intensified (N= 1,262)**	**SMD**
Age, mean (SD), y	71.8 (7)	73.3 (7.7)	0.21	71.9 (7.1)	71.8 (6.8)	0.02
Male sex, No. (%)	1323 (98)	5633 (98.6)	0.05	1238 (98.1)	1240 (98.3)	0.01
Race/ethnicity, No. (%)			0.12			0.03
White	1037 (76.8)	4582 (80.2)		973 (77.1)	990 (78.4)	
Black	237 (17.6)	805 (14.1)		216 (17.1)	201 (15.9)	
Hispanic	11 (0.8)	92 (1.6)		10 (0.8)	9 (0.7)	
Other	65 (4.8)	232 (4.1)		63 (5)	62 (4.9)	
Median zip code household income, mean (SD), $	26194 (44060.3)	27584.5 (50133.9)	0.03	26522.7 (45380)	26171.1 (38143.1)	0.01
VISN, No. (%)			0.20			0.11
New England Health Care System	50 (3.7)	301 (5.3)		50 (4)	52 (4.1)	
Healthcare New York/New Jersey	71 (5.3)	292 (5.1)		68 (5.4)	81 (6.4)	
VISN4 Network	62 (4.6)	253 (4.4)		56 (4.4)	49 (3.9)	
Capitol Healthcare Network	31 (2.3)	146 (2.6)		30 (2.4)	35 (2.8)	
Mid-Atlantic Health Care Network	74 (5.5)	316 (5.5)		73 (5.8)	87 (6.9)	
Southeast Network	90 (6.7)	259 (4.5)		82 (6.5)	84 (6.7)	
Sunshine Healthcare Network	99 (7.3)	538 (9.4)		97 (7.7)	94 (7.4)	
MidSouth Healthcare Network	74 (5.5)	431 (7.5)		68 (5.4)	65 (5.2)	
VISN 10 Healthcare System	49 (3.6)	216 (3.8)		46 (3.6)	45 (3.6)	
Great Lakes Healthcare System	130 (9.6)	505 (8.8)		119 (9.4)	112 (8.9)	
Heartland Network	69 (5.1)	276 (4.8)		66 (5.2)	57 (4.5)	
South Central VA Healthcare Network	171 (12.7)	553 (9.7)		156 (12.4)	152 (12)	
Heart of Texas Healthcare System	63 (4.7)	303 (5.3)		58 (4.6)	65 (5.2)	
Southwest Healthcare Network	53 (3.9)	185 (3.2)		45 (3.6)	36 (2.9)	
Rocky Mountain Network	33 (2.4)	140 (2.5)		30 (2.4)	28 (2.2)	
Northwest Health Network	43 (3.2)	208 (3.6)		38 (3)	29 (2.3)	
Sierra Pacific Network	58 (4.3)	269 (4.7)		56 (4.4)	56 (4.4)	
Desert Pacific Healthcare Network	71 (5.3)	294 (5.1)		66 (5.2)	78 (6.2)	
Midwest Healthcare Network	59 (4.4)	226 (4)		58 (4.6)	57 (4.5)	
**Pre-admission clinical characteristics, mean (SD)**						
Body mass index, kg/m^2	31.4 (6.5)	31.2 (6.6)	0.04	31.4 (6.3)	31.5 (6.9)	0.02
Systolic BP, mmHg	135.8 (18.2)	134.6 (17.5)	0.07	135.7 (18.1)	136.3 (18)	0.03

© 2021 Anderson TS et al. *JAMA Network Open.*

Diastolic BP, mmHg	74.7 (10.4)	73.5 (10.1)	0.11	74.5 (10.4)	74.6 (10.7)	0.00
Frailty score	0.2 (0.1)	0.2 (0.1)	0.03	0.2 (0.1)	0.2 (0.1)	0.01
**Pre-admission diabetes management**						
Hemoglobin A1c, mean (SD), d	9.2 (1.6)	8.6 (1.2)	0.43	9.1 (1.5)	9.1 (1.6)	0.01
Estimated glomerular filtration rate, mean (SD), mL/min per 1.73 m2	66.1 (23.8)	66.7 (24)	0.03	66.1 (23.7)	65.7 (25.4)	0.02
Any hypoglycemia hospitalizations in prior year, No. (%)	16 (1.2)	62 (1.1)	0.01	14 (1.1)	15 (1.2)	0.01
Number of admission diabetes medications, No. (%)			0.28			0.03
0	566 (41.9)	1701 (29.8)		507 (40.2)	496 (39.3)	
1	448 (33.2)	2003 (35.1)		423 (33.5)	420 (33.3)	
2	284 (21)	1709 (29.9)		281 (22.3)	290 (23)	
>3	52 (3.9)	298 (5.2)		51 (4)	56 (4.5)	
Admission diabetes medication classes, No. (%) [b]						
Metformin	513 (38)	2670 (46.8)	0.18	493 (39.1)	489 (38.7)	0.01
Sulfonylureas	543 (40.2)	3081 (53.9)	0.28	532 (42.2)	555 (44)	0.04
Thiazolidinediones	45 (3.3)	220 (3.9)	0.03	44 (3.5)	54 (4.3)	0.04
α-glucosidase inhibitors	42 (3.1)	131 (2.3)	0.05	41 (3.2)	49 (3.9)	0.03
Dipeptidyl peptidase-4 inhibitors	26 (1.9)	163 (2.9)	0.06	25 (2)	24 (1.9)	0.01
Other classes	2 (0.1)	21 (0.4)	0.04	2 (0.2)	1 (0.1)	0.02
Preadmission diabetes medication adherence			0.26			0.02
0 medications	566 (41.9)	1701 (29.8)		507 (40.2)	496 (39.3)	
PDC ≤ 80%	321 (23.8)	1540 (27)		309 (24.5)	314 (24.9)	
PDC > 80%	463 (34.3)	2470 (43.2)		446 (35.3)	452 (35.8)	
Diabetes medication classes used in prior year, No. (%)						
Metformin	712 (52.7)	3045 (53.3)	0.01	659 (52.2)	662 (52.5)	0.00
Sulfonylureas	721 (53.4)	3406 (59.6)	0.13	687 (54.4)	705 (55.9)	0.03
Thiazolidinediones	61 (4.5)	286 (5)	0.02	59 (4.7)	70 (5.5)	0.04
α-glucosidase inhibitors	54 (4)	160 (2.8)	0.07	51 (4)	57 (4.5)	0.02
Dipeptidyl peptidase-4 inhibitors	19 (1.4)	105 (1.8)	0.03	19 (1.5)	14 (1.1)	0.03
Insulins	416 (30.8)	635 (11.1)	0.50	348 (27.6)	358 (28.4)	0.02
Other classes	5 (0.4)	33 (0.6)	0.03	5 (0.4)	4 (0.3)	0.01
**Pre-admission healthcare use, No. (%)**						
Any Medicare utilization in prior year, No. (%)	378 (28)	1724 (30.2)	0.05	357 (28.3)	367 (29.1)	0.02
No. ED visits in the year preceding index hospitalization			0.04			0.04
0	696 (51.6)	2908 (50.9)		656 (52)	671 (53.2)	
1	314 (23.3)	1348 (23.6)		288 (22.8)	292 (23.1)	
2	168 (12.4)	659 (11.5)		155 (12.3)	151 (12)	

© 2021 Anderson TS et al. *JAMA Network Open.*

>3	172 (12.7)	796 (13.9)		163 (12.9)	148 (11.7)	
No. hospitalizations in the year preceding index hospitalization			0.05			0.04
0	956 (70.8)	4153 (72.7)		904 (71.6)	884 (70)	
1	241 (17.9)	956 (16.7)		220 (17.4)	225 (17.8)	
2	93 (6.9)	340 (6)		83 (6.6)	95 (7.5)	
>3	60 (4.4)	262 (4.6)		55 (4.4)	58 (4.6)	
Admission medication count, mean (SD)	7.2 (4.7)	7.7 (5.2)	0.10	7.3 (4.7)	7.2 (4.9)	0.01
Polypharmacy (>8 medications), No. (%)	637 (47.2)	2974 (52.1)	0.10	607 (48.1)	602 (47.7)	0.01
**Index hospitalization characteristics**						
Length of stay, mean (SD), d	6.5 (8.5)	5.2 (5.6)	0.18	6.1 (6.6)	6.4 (8.9)	0.04
Year of hospitalization, No. (%)			0.08			0.06
2011	270 (20)	1171 (20.5)		251 (19.9)	269 (21.3)	
2012	223 (16.5)	1011 (17.7)		207 (16.4)	211 (16.7)	
2013	210 (15.6)	923 (16.2)		203 (16.1)	208 (16.5)	
2014	226 (16.7)	910 (15.9)		210 (16.6)	189 (15)	
2015	213 (15.8)	949 (16.6)		199 (15.8)	185 (14.7)	
2016	208 (15.4)	747 (13.1)		192 (15.2)	200 (15.8)	
Training hospital, No. (%)	1197 (88.7)	5141 (90)	0.04	1123 (89)	1129 (89.5)	0.02
**Discharge diagnoses, No. (%)**			0.16			0.09
Arrhythmia	12 (0.9)	80 (1.4)		12 (1)	13 (1)	
Asthma	10 (0.7)	18 (0.3)		8 (0.6)	6 (0.5)	
Chronic obstructive pulmonary disease	95 (7)	379 (6.6)		89 (7.1)	97 (7.7)	
Chest pain	40 (3)	194 (3.4)		39 (3.1)	36 (2.9)	
Conduction disorders	96 (7.1)	569 (10)		95 (7.5)	89 (7.1)	
Coronary artery disease	181 (13.4)	665 (11.6)		170 (13.5)	159 (12.6)	
Acute coronary syndrome	93 (6.9)	420 (7.4)		87 (6.9)	82 (6.5)	
Heart failure	236 (17.5)	856 (15)		216 (17.1)	219 (17.4)	
Heart valve disorders	24 (1.8)	104 (1.8)		24 (1.9)	23 (1.8)	
Pneumonia	124 (9.2)	583 (10.2)		119 (9.4)	120 (9.5)	
Sepsis	39 (2.9)	176 (3.1)		31 (2.5)	29 (2.3)	
Skin infection	138 (10.2)	555 (9.7)		127 (10.1)	112 (8.9)	
Stroke	105 (7.8)	394 (6.9)		97 (7.7)	115 (9.1)	
Transient ischemic attack	17 (1.3)	92 (1.6)		15 (1.2)	20 (1.6)	
Urinary tract infection	103 (7.6)	480 (8.4)		99 (7.8)	101 (8)	
Venous thromboembolism	37 (2.7)	146 (2.6)		34 (2.7)	41 (3.2)	
**Hospital blood glucose values, mean (SD)**						
Highest glucose	335.5 (111.3)	280.5 (89.5)	0.55	326.5 (104.8)	328.2 (102.9)	0.02
Lowest glucose	112.5 (50)	111.9 (41.3)	0.01	113.4 (48.2)	114.5 (47.4)	0.02
Standard deviation of hospital glucose recordings	63.4 (30.6)	50.2 (25.6)	0.47	61.1 (29.1)	61.3 (29.9)	0.01
**Laboratory values at discharge, mean (SD)** [c]						
Glucose, mg/dL	193.6 (74.5)	184.5 (66.4)	0.13	193.7 (73.7)	196.4 (73.4)	0.04
Hemoglobin, g/dL	12.1 (2)	12.2 (1.9)	0.04	12.2 (2)	12.2 (1.9)	0.01

© 2021 Anderson TS et al. *JAMA Network Open.*

Platelets, k/mm3	225.1 (85)	217 (82.7)	0.10	224.1 (84.2)	227.5 (93.9)	0.04
Sodium, mEQ/L	137.6 (3.1)	137.7 (3.1)	0.04	137.5 (3.2)	137.5 (3.1)	0.00
Potassium, mEQ/L	4.1 (0.4)	4.1 (0.4)	0.01	4.1 (0.4)	4.1 (0.4)	0.02
Blood urea nitrogen, mg/dL	24.3 (14.6)	23.1 (13.5)	0.08	24.2 (14.5)	24.5 (14.7)	0.02
Carbon dioxide, mEQ/L	27 (3.8)	26.8 (3.8)	0.05	27 (3.8)	27 (3.9)	0.00
Estimated glomerular filtration rate, mL/min per 1.73 m2	67.5 (26.4)	69 (27.5)	0.06	67.7 (26.3)	67.7 (30.8)	0.00
**Other medication classes used at hospital admission, No. (%)**[b]						
Antihistamines	142 (10.5)	693 (12.1)	0.05	133 (10.5)	123 (9.7)	0.03
Antimicrobials	47 (3.5)	166 (2.9)	0.03	46 (3.6)	32 (2.5)	0.06
Antineoplastics	26 (1.9)	116 (2)	0.01	24 (1.9)	24 (1.9)	0.00
Autonomic medications	13 (1)	89 (1.6)	0.05	13 (1)	13 (1)	0.00
Anticoagulants	134 (9.9)	758 (13.3)	0.10	125 (9.9)	134 (10.6)	0.02
Platelet aggregation inhibitors	119 (8.8)	573 (10)	0.04	115 (9.1)	116 (9.2)	0.00
Other central nervous system medications	94 (7)	408 (7.1)	0.01	85 (6.7)	94 (7.4)	0.03
Non-opioid analgesics	366 (27.1)	1553 (27.2)	0.00	340 (26.9)	326 (25.8)	0.03
Opioid analgesics	233 (17.3)	991 (17.4)	0.00	220 (17.4)	222 (17.6)	0.00
Sedative/hypnotics	109 (8.1)	525 (9.2)	0.04	106 (8.4)	104 (8.2)	0.01
Anticonvulsants	250 (18.5)	918 (16.1)	0.06	229 (18.1)	211 (16.7)	0.04
Antdepressants	314 (23.3)	1273 (22.3)	0.02	292 (23.1)	301 (23.9)	0.02
Antipsychotics	37 (2.7)	200 (3.5)	0.04	36 (2.9)	35 (2.8)	0.00
Digitalis glycosides	47 (3.5)	266 (4.7)	0.06	43 (3.4)	41 (3.2)	0.01
Beta blockers	629 (46.6)	2709 (47.4)	0.02	583 (46.2)	591 (46.8)	0.01
Alpha blockers	321 (23.8)	1442 (25.2)	0.03	304 (24.1)	300 (23.8)	0.01
Calcium channel blockers	365 (27)	1499 (26.2)	0.02	345 (27.3)	346 (27.4)	0.00
Antianginals	214 (15.9)	856 (15)	0.02	203 (16.1)	186 (14.7)	0.04
Antiarrhythmics	21 (1.6)	86 (1.5)	0.00	20 (1.6)	20 (1.6)	0.00
Antilipemics	830 (61.5)	3520 (61.6)	0.00	775 (61.4)	779 (61.7)	0.01
Other antihypertensives	125 (9.3)	513 (9)	0.01	120 (9.5)	124 (9.8)	0.01
Diuretics	256 (19)	1062 (18.6)	0.01	238 (18.9)	266 (21.1)	0.06
Loop diuretics	307 (22.7)	1304 (22.8)	0.00	285 (22.6)	288 (22.8)	0.01
Angiotension converting enzyme inhibitors	539 (39.9)	2217 (38.8)	0.02	499 (39.5)	498 (39.5)	0.00
Angiotensin II receptor blockers	169 (12.5)	729 (12.8)	0.01	159 (12.6)	157 (12.4)	0.00
Gastrointenstinal medications	540 (40)	2399 (42)	0.04	508 (40.3)	494 (39.1)	0.02
Genitourinary medications	126 (9.3)	628 (11)	0.06	123 (9.7)	112 (8.9)	0.03
Hormones	277 (20.5)	1284 (22.5)	0.05	257 (20.4)	248 (19.7)	0.02
Musculoskeletal medications	253 (18.7)	1032 (18.1)	0.02	237 (18.8)	240 (19)	0.01
Antiasthma/brochodilators	13 (1)	87 (1.5)	0.05	13 (1)	17 (1.3)	0.03
Systemic decongestants and antitussives	45 (3.3)	229 (4)	0.04	42 (3.3)	49 (3.9)	0.03
**Comorbidities, No. (%)** [d]						
Heart failure	507 (37.6)	2012 (35.2)	0.05	469 (37.2)	473 (37.5)	0.01
Acute myocardial infarction	133 (9.9)	604 (10.6)	0.02	124 (9.8)	126 (10)	0.01
Unstable angina and other acute ischemic heart disease	85 (6.3)	411 (7.2)	0.04	78 (6.2)	81 (6.4)	0.01

© 2021 Anderson TS et al. *JAMA Network Open.*

Chronic angina and coronary artery disease	664 (49.2)	2969 (52)	0.06	619 (49)	603 (47.8)	0.03
Valvular/rheumatic heart disease	168 (12.4)	817 (14.3)	0.05	160 (12.7)	160 (12.7)	0.00
Other cardiac disease including congenital heart and hypertensive disease	801 (59.3)	3497 (61.2)	0.04	742 (58.8)	741 (58.7)	0.00
Arrhythmias and conduction disorders	572 (42.4)	2719 (47.6)	0.11	539 (42.7)	561 (44.5)	0.04
Pleural effusion/pneumothorax	86 (6.4)	443 (7.8)	0.05	82 (6.5)	88 (7)	0.02
Chest pain	312 (23.1)	1433 (25.1)	0.05	301 (23.9)	294 (23.3)	0.01
Syncope	75 (5.6)	342 (6)	0.02	70 (5.5)	82 (6.5)	0.04
Acute stroke/transient ischemic attack	242 (17.9)	996 (17.4)	0.01	218 (17.3)	231 (18.3)	0.03
Pulmonary embolism/deep venous thrombosis	86 (6.4)	375 (6.6)	0.01	80 (6.3)	77 (6.1)	0.01
Other peripheral vascular disease	297 (22)	1199 (21)	0.02	272 (21.6)	283 (22.4)	0.02
Cardio-respiratory failure	30 (2.2)	127 (2.2)	0.00	26 (2.1)	29 (2.3)	0.02
Chronic obstructive pulmonary disease/asthma	500 (37)	2202 (38.6)	0.03	469 (37.2)	456 (36.1)	0.02
Pneumonia including aspiration pneumonitis	269 (19.9)	1136 (19.9)	0.00	249 (19.7)	267 (21.2)	0.04
Septicemia/shock	91 (6.7)	406 (7.1)	0.01	84 (6.7)	89 (7.1)	0.02
Urinary tract infection and urinary system complaints	251 (18.6)	1071 (18.8)	0.00	234 (18.5)	227 (18)	0.01
Cellulitis	283 (21)	1137 (19.9)	0.03	259 (20.5)	258 (20.4)	0.00
Clostridium difficile-associated infection	13 (1)	54 (0.9)	0.00	11 (0.9)	14 (1.1)	0.02
Renal disorders including renal failure and fluid, electrolyte, and acid-base abnormalities	688 (51)	2768 (48.5)	0.05	627 (49.7)	636 (50.4)	0.01
Anemia	402 (29.8)	1670 (29.2)	0.01	371 (29.4)	378 (30)	0.01
Gastrointestinal hemorrhage	80 (5.9)	372 (6.5)	0.02	75 (5.9)	70 (5.5)	0.02
Fibrosis of lung and other chronic lung disorders	29 (2.1)	177 (3.1)	0.06	27 (2.1)	27 (2.1)	0.00
Hip fracture	10 (0.7)	35 (0.6)	0.02	9 (0.7)	14 (1.1)	0.04
Complications of care	161 (11.9)	788 (13.8)	0.06	149 (11.8)	141 (11.2)	0.02
Other lung disorders including acute, congenital, and unspecified lung abnormalities	495 (36.7)	2313 (40.5)	0.08	469 (37.2)	472 (37.4)	0.00
Primary cancer of the trachea, bronchus, lung, and pleura	26 (1.9)	144 (2.5)	0.04	23 (1.8)	25 (2)	0.01
Other infections	664 (49.2)	2957 (51.8)	0.05	619 (49)	616 (48.8)	0.00
Cancer	235 (17.4)	1191 (20.9)	0.09	223 (17.7)	209 (16.6)	0.03
Endocrine/metabolic	1255 (93)	5364 (93.9)	0.04	1176 (93.2)	1186 (94)	0.03
Liver disease	112 (8.3)	474 (8.3)	0.00	103 (8.2)	100 (7.9)	0.01
Digestive (Gastrointestinal)	702 (52)	3238 (56.7)	0.09	663 (52.5)	670 (53.1)	0.01
Musculoskeletal	990 (73.3)	4390 (76.9)	0.08	924 (73.2)	901 (71.4)	0.04
Other hematologic	181 (13.4)	744 (13)	0.01	167 (13.2)	168 (13.3)	0.00

© 2021 Anderson TS et al. *JAMA Network Open.*

Cognitive	142 (10.5)	717 (12.6)	0.06	131 (10.4)	139 (11)	0.02
Substance abuse	151 (11.2)	473 (8.3)	0.10	131 (10.4)	141 (11.2)	0.03
Psychiatric	607 (45)	2455 (43)	0.04	562 (44.5)	564 (44.7)	0.00
Development disorders	16 (1.2)	62 (1.1)	0.01	13 (1)	10 (0.8)	0.03
Spinal cord	37 (2.7)	171 (3)	0.02	35 (2.8)	38 (3)	0.01
Neuromuscular	698 (51.7)	2844 (49.8)	0.04	644 (51)	636 (50.4)	0.01
Non-acute cerebrovascular disease	174 (12.9)	835 (14.6)	0.05	165 (13.1)	155 (12.3)	0.02
Other pulmonary	28 (2.1)	125 (2.2)	0.01	27 (2.1)	33 (2.6)	0.03
Ophthalmologic	1031 (76.4)	4520 (79.1)	0.07	965 (76.5)	955 (75.7)	0.02
Ear, nose, and throat	535 (39.6)	2747 (48.1)	0.17	510 (40.4)	504 (39.9)	0.01
Other renal	230 (17)	1004 (17.6)	0.01	206 (16.3)	224 (17.7)	0.04
Genitourinary	667 (49.4)	2855 (50)	0.01	623 (49.4)	617 (48.9)	0.01
Other dermatologic	614 (45.5)	2663 (46.6)	0.02	568 (45)	589 (46.7)	0.03
Injury excluding hip fracture	294 (21.8)	1350 (23.6)	0.04	279 (22.1)	265 (21)	0.03
Symptoms (major and minor) excluding respiratory	492 (36.4)	2173 (38)	0.03	452 (35.8)	458 (36.3)	0.01
Miscellaneous surgical and non-surgical procedures	1318 (97.6)	5593 (97.9)	0.02	1231 (97.5)	1221 (96.8)	0.05
Durable medical equipment	357 (26.4)	1626 (28.5)	0.05	335 (26.5)	335 (26.5)	0.00
Other	1038 (76.9)	4556 (79.8)	0.07	970 (76.9)	957 (75.8)	0.02
Skin ulcer including decubitus ulcer	175 (13)	683 (12)	0.03	160 (12.7)	162 (12.8)	0.00

© 2021 Anderson TS et al. *JAMA Network Open.*

eTable 7. Secondary Clinical Outcomes Associated with Receiving Diabetes Medication Intensifications at Hospital Discharge in Subgroups with Controlled and Elevated Prehospitalization Hemoglobin A_{1c} Subgroups

	Controlled Baseline HbA_{1c} Subgroup ($HbA_{1c} \leq 7.5\%$)			Elevated Baseline HbA_{1c} Subgroup ($HbA_{1c} > 7.5\%$)		
	No. (%) of Patients			No. (%) of Patients		
	Intensified (N=1,336)	Not Intensified (N=1,336)	Hazard Ratio (95% CI)	Intensified (N=1,262)	Not Intensified (N=1,262)	Hazard Ratio (95% CI)
**Secondary outcomes**						
**Mortality**						
30 days	21 (1.6)	33 (2.5)	0.63 (0.35 to 1.15)	14 (1.1)	30 (2.4)	0.46 (0.24 to 0.91)
365 days	236 (17.7)	249 (18.6)	0.93 (0.78 to 1.11)	117 (14.0)	231 (18.3)	0.75 (0.62 to 0.89)
**Readmission**						
30 days	242 (18.1)	258 (19.3)	0.93 (0.78 to 1.10)	210 (16.6)	209 (16.6)	1.01 (0.83 to 1.23)
365 days	729 (54.6)	720 (53.9)	1.01 (0.92 to 1.10)	630 (49.9)	622 (4.3)	1.03 (0.92 to 1.15)

© 2021 Anderson TS et al. *JAMA Network Open.*

eTable 8. Difference in Differences Analysis of Hemoglobin A_{1c} Values One Year Following Discharge With or Without Diabetes Medication Intensifications in Controlled and Elevated Baseline HbA_{1c} Subgroups

	Controlled Baseline HbA_{1c} Subgroup		Elevated Baseline HbA_{1c} Subgroup	
**HbA_{1c}, Mean (95% CI), % [a]**	**Intensified Regimen (N=1,071)**	**Not Intensified Regimen (N=1,027)**	**Intensified Regimen (N=1,051)**	**Not Intensified Regimen (N=983)**
Baseline	6.84 (6.78 to 6.90)	6.83 (6.77 to 6.89)	9.06 (8.96 to 9.15)	9.00 (8.89 to 9.10)
Post-baseline [b]	7.19 (7.14 to 7.25)	7.16 (7.10 to 7.22)	8.26 (8.16 to 8.36)	8.36 (8.26 to 8.47)
Pre-post change	0.36 (0.28 to 0.44)	0.33 (0.25 to 0.41)	-0.79 (-0.93 to -0.65)	-0.63 (-0.78 to -0.49)
Difference-in-differences estimate [c]	0.03 (-0.09 to 0.14)		-0.16 (-0.36 to 0.04)	

Abbreviations: SD, standard deviation, HbA_{1c}, hemoglobin A1c

[a] The results are from patients in each propensity-matched cohort who met the inclusion criteria for the HbA_{1c} analysis (outpatient HbA_{1c} measured between 6 months and 18 months following index hospitalization discharge). Mean (SD) number of days between baseline and postbaseline measures for controlled baseline HbA_{1c} subgroup: 281 (80) days for intensified group and 283 (84) days for not intensified group. Mean (SD) number of days between baseline and postbaseline measures for elevated baseline HbA_{1c} subgroup: 274 (78) days for intensified group and 276 (83) days for not intensified group.

[b] The post-discharge HbA_{1c} was assessed prior to censoring as the HbA_{1c} recorded at the closest date to 1 year following index hospitalization discharge, within the range for 6 to 18 months following discharge.

[c] Calculated as the pre-post change in HbA_{1c} for the intensified regimen group minus the pre-post change in HbA_{1c} for the not intensified group.

© 2021 Anderson TS et al. *JAMA Network Open.*

eFigure 1. Flow Diagram of Underlying Study Population

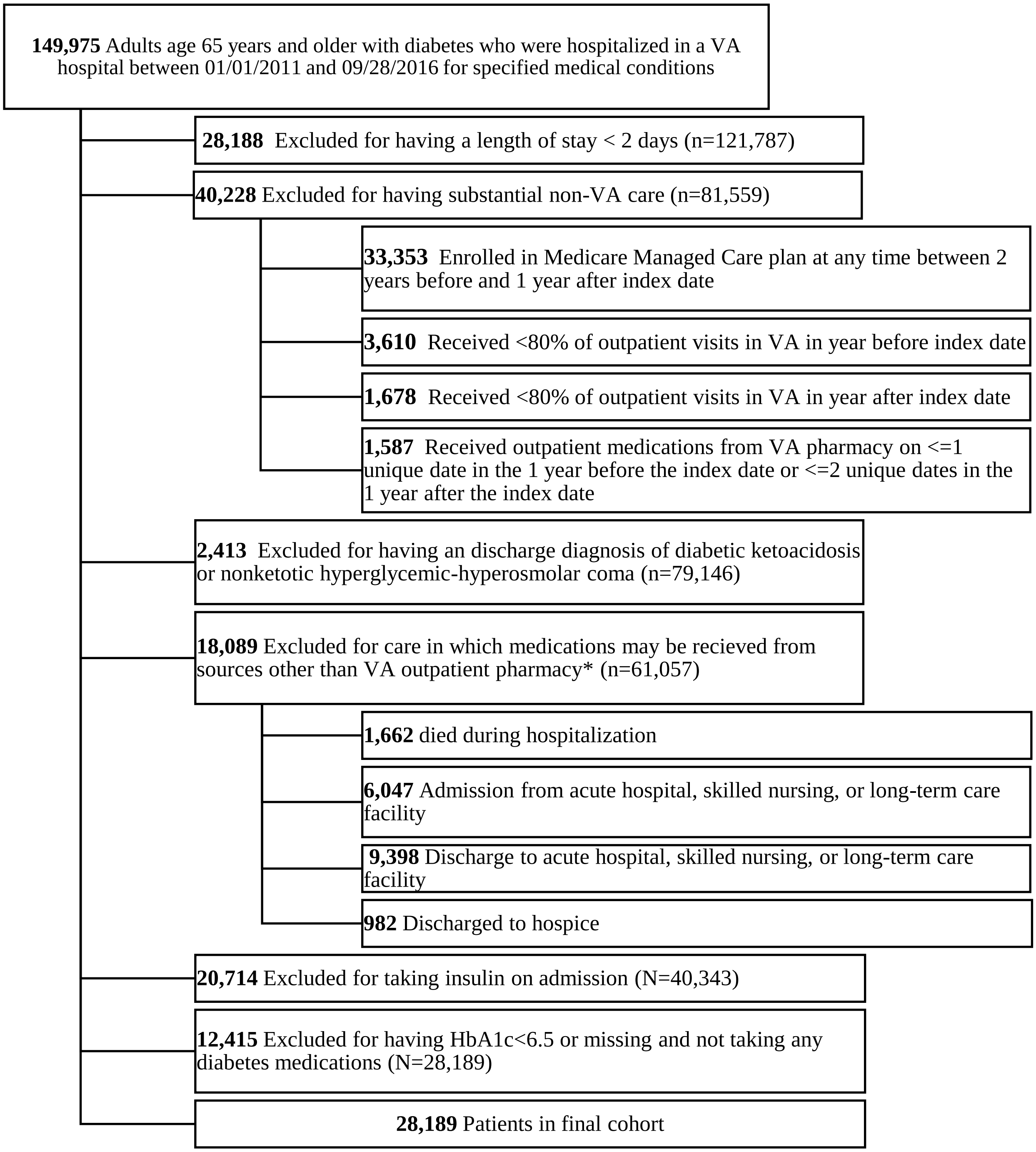

© 2021 Anderson TS et al. *JAMA Network Open.*

eFigure 2. Propensity Score Distributions Before and After Matching

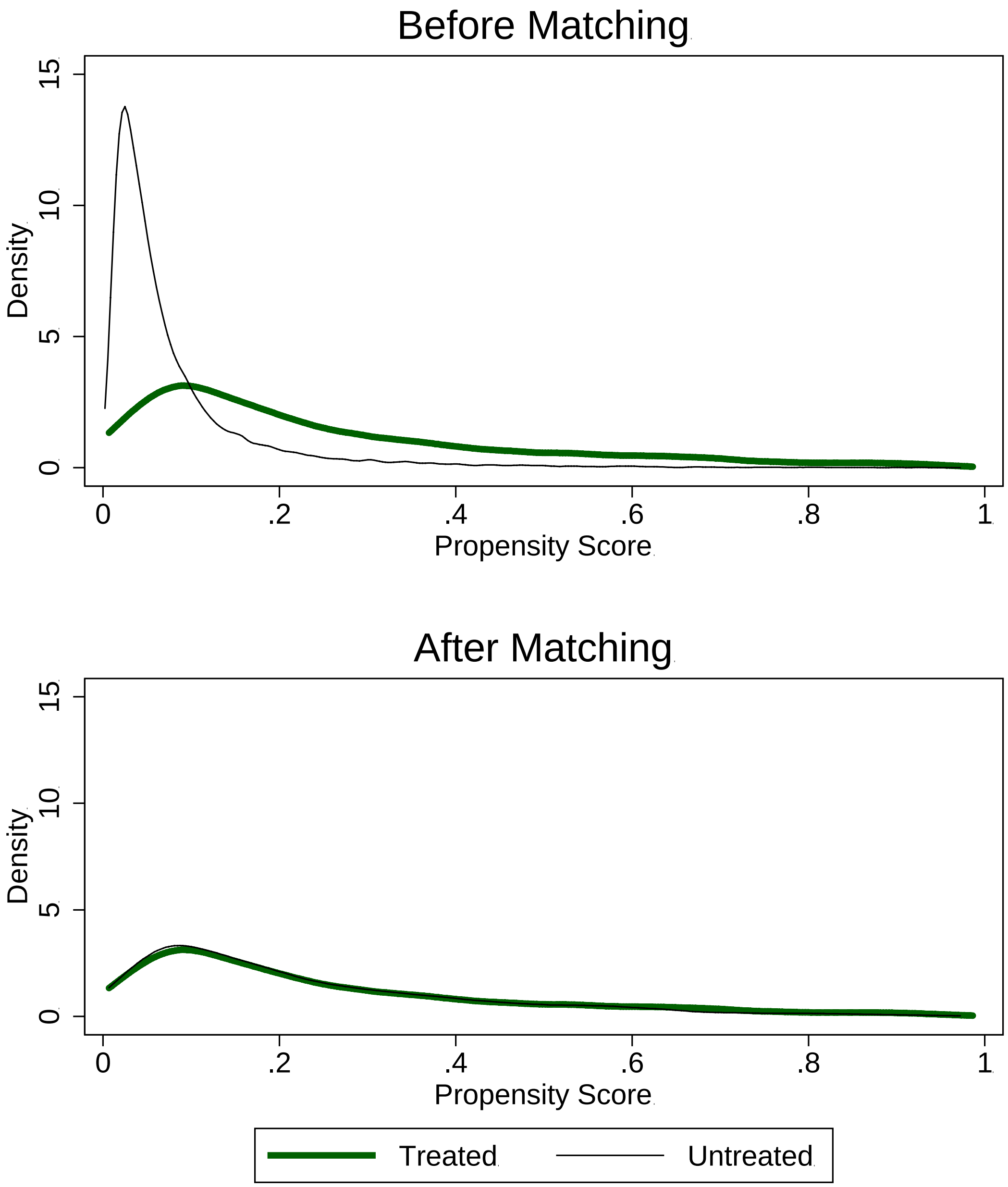

© 2021 Anderson TS et al. *JAMA Network Open.*

eFigure 3. Propensity Score Distributions Before and After Matching in Controlled and Elevated Baseline HbA_{1c} Subgroups

A) Controlled HbA_{1c} Subgroup

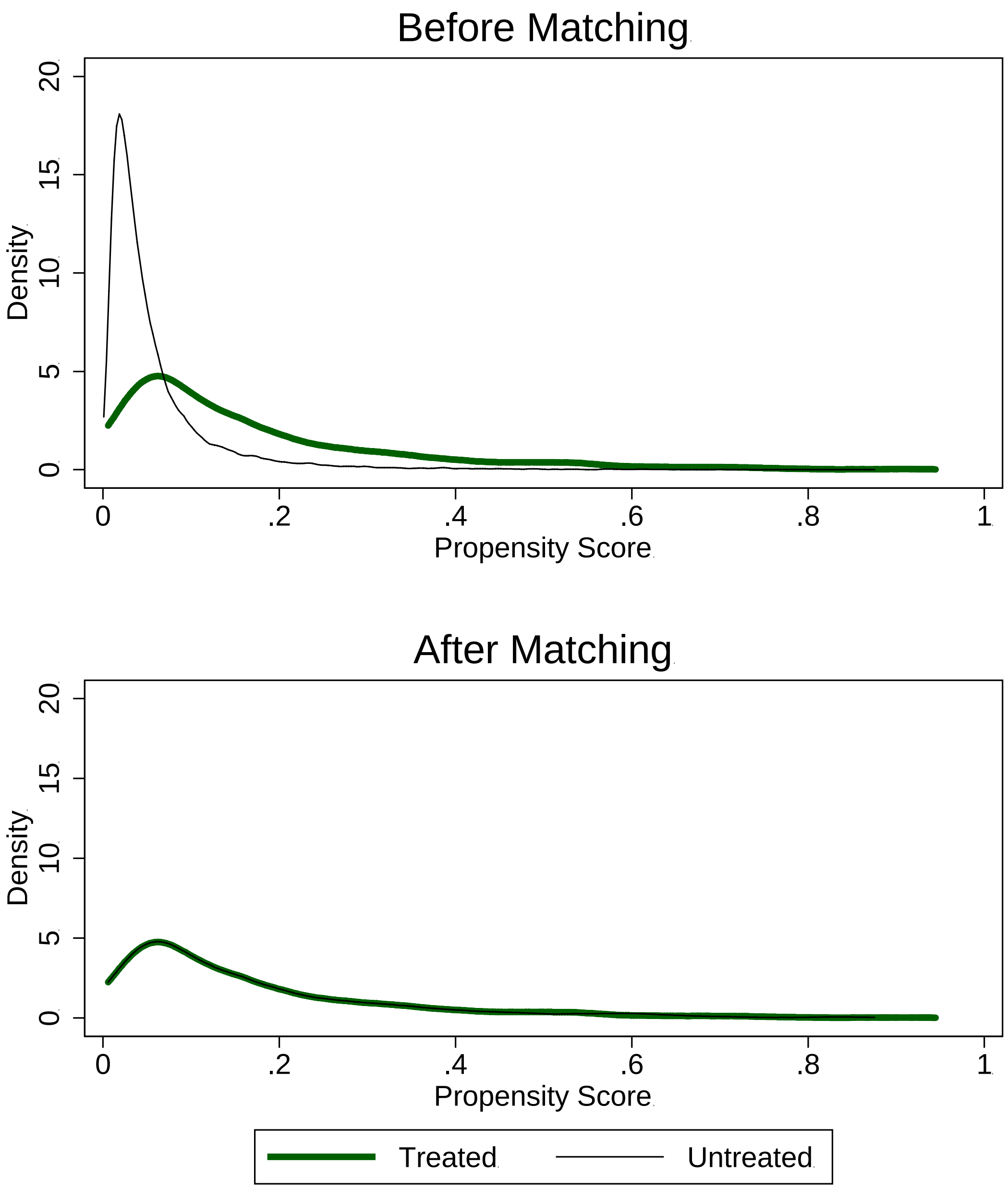

© 2021 Anderson TS et al. *JAMA Network Open.*

B) Elevated Hb_{A1c} Subgroup

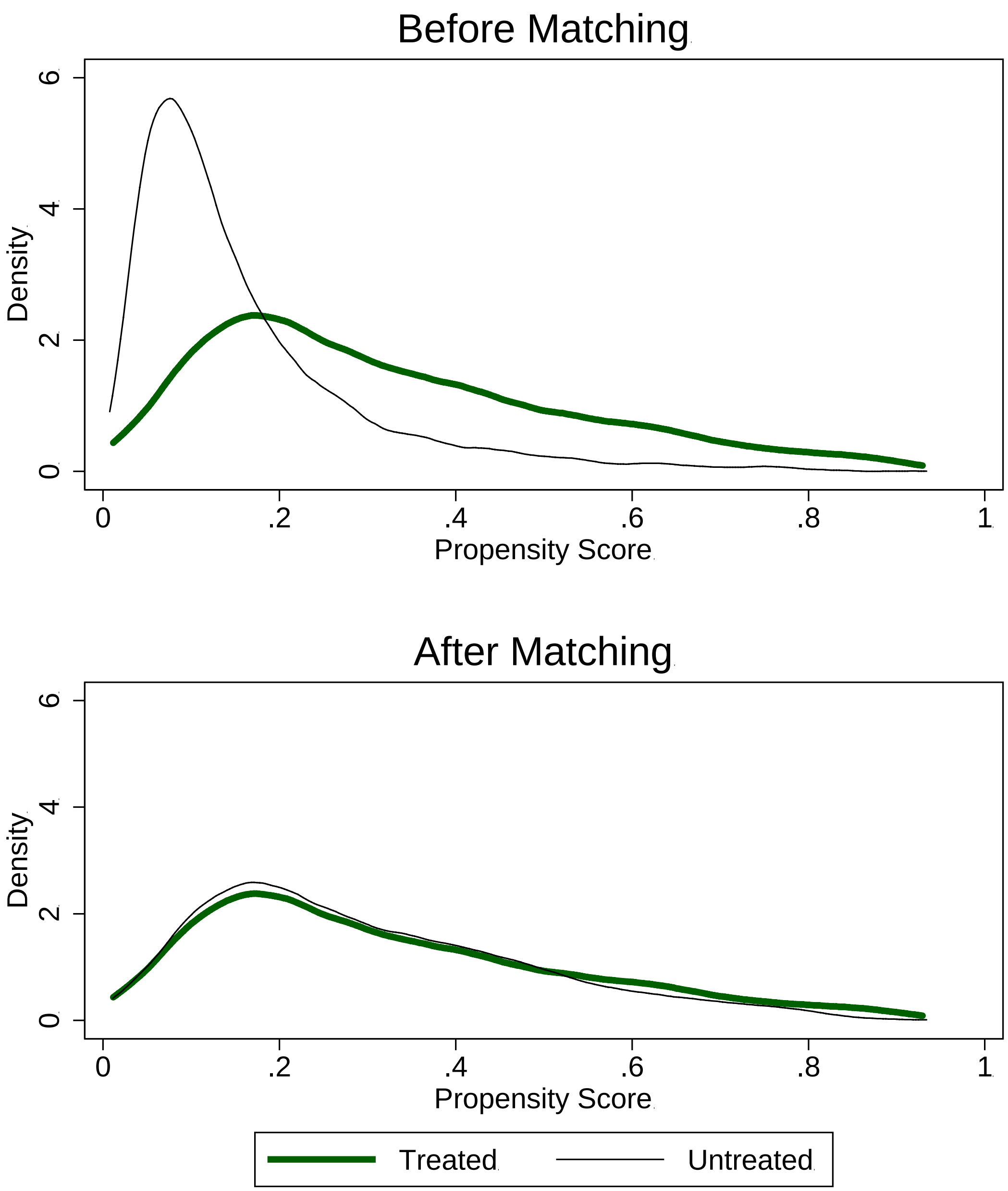

© 2021 Anderson TS et al. *JAMA Network Open.*
